# T2DiACoD: A Gene Atlas of Type 2 Diabetes Mellitus Associated Complex Disorders

**DOI:** 10.1038/s41598-017-07238-0

**Published:** 2017-07-31

**Authors:** Jyoti Rani, Inna Mittal, Atreyi Pramanik, Namita Singh, Namita Dube, Smriti Sharma, Bhanwar Lal Puniya, Muthukurussi Varieth Raghunandanan, Ahmed Mobeen, Srinivasan Ramachandran

**Affiliations:** 1 G N Ramachandran Knowledge of Centre, Council of Scientific and Industrial Research – Institute of Genomics and Integrative Biology (CSIR-IGIB), Room No. 130, Mathura Road, New Delhi, 110025 India; 2 Academy of Scientific and Innovative Research, CSIR-IGIB South Campus, New Delhi, 110025 India

## Abstract

We performed integrative analysis of genes associated with type 2 Diabetes Mellitus (T2DM) associated complications by automated text mining with manual curation and also gene expression analysis from Gene Expression Omnibus. They were analysed for pathogenic or protective role, trends, interaction with risk factors, Gene Ontology enrichment and tissue wise differential expression. The database T2DiACoD houses 650 genes, and 34 microRNAs associated with T2DM complications. Seven genes *AGER, TNFRSF11B, CRK, PON1, ADIPOQ, CRP* and *NOS3* are associated with all 5 complications. Several genes are studied in multiple years in all complications with high proportion in cardiovascular (75.8%) and atherosclerosis (51.3%). T2DM Patients’ skeletal muscle tissues showed high fold change in differentially expressed genes. Among the differentially expressed genes, *VEGFA* is associated with several complications of T2DM. A few genes *ACE2*, *ADCYAP1*, *HDAC4*, *NCF1*, *NFE2L2*, *OSM*, *SMAD1*, *TGFB1*, *BDNF*, *SYVN1*, *TXNIP*, *CD36*, *CYP2J2*, *NLRP3* with details of protective role are catalogued. Obesity is clearly a dominant risk factor interacting with the genes of T2DM complications followed by inflammation, diet and stress to variable extents. This information emerging from the integrative approach used in this work could benefit further therapeutic approaches. The T2DiACoD is available at www.http://t2diacod.igib.res.in/.

## Introduction

T2DM is on the rise^1^. It is characterized by hyperglycaemia due to insulin resistance and decreased insulin activity. Genetic factors, environmental agents and their interactions have been recognized as contributors to the development of T2DM^2–4^. As of 2014, more than 371 million people reportedly suffer from T2DM in several countries with China at the top (92.3 million) followed by India (80 million) and the USA (29.1 million)^5^. Before the era of genome wide association studies (GWAS), hunting for genetic factors produced a handful of genes such as *CAPN10*, *TCF7L2, PPARG* and *KCNJ11* ^6, 7^. The association of *TCF7L2, PPARG* and *KCNJ11* with T2DM have been observed in several populations and both PPARG and KCNJ11 proteins are targets of currently used T2DM drugs^7^. The GWAS era including meta-analysis enabled uncovering of numerous genes and their variants associated with T2DM. Yet these variants apparently are able to explain only about 20–30% of heritable component^8^. Re-sequencing is now emerging to enable comprehensive variants discovery with its identified potential not offered by imputation and chip based fine-mapping approaches^8^. These efforts can get further challenged by gene environment interactions whereby phenotypic effects for given genotypes likely vary^9^. Diet and physical activity are most considered among environmental factors. Dietary components could affect directly (through interaction with targets) and indirectly (through interaction with gut microbiome). Although some compounds such as vitamins and organic pollutants such as heptachlor epoxide and polychlorinated biphenyls have been studied, this area is relatively under investigated and most environmental agents are not known^5, 10^. Despite these deficiencies the huge body of studies carried out so far including those in animal models have endowed us with many genes associated with T2DM and its associated complications that can serve for identifying drug targets, potential biomarkers and clinical applications.

Several GWAS have been carried out to identify genetic susceptibility to T2DM in different populations^11–14^. Recently Fuchsberger *et al*. analysed the T2DM associated variants by increasing the sample size and observed that most of the variants were located within the same regions previously identified by GWAS^14^. Studies including meta-analysis enabled identification of 44 genetic loci associated independently with transcriptome data sets of human tissues from T2DM patients. These studies are valuable for gaining insights into the probable pathways leading to T2DM^15, 16^. Integration of genes co-expression and interaction networks enabled identification of TGFBRII, MAPK, PTPN1, EGFR, and CAV1 pathways, which could lead to cardiovascular and kidney related complications and diabetic nephropathy and Zelezniak *et al*. integrated skeletal muscle gene expression datasets with human metabolic network reconstructions, which enabled them to identify transcription factors CREB, NRF1 and PPAR family regulatory network connecting several parts of metabolism^17, 18^. Zhong *et al*. (2010)^19^ identified a functionally related set of diabetes susceptibility genes using expression SNPs (eSNPs) comprising an adipose sub-network and subsequently validated malic enzyme (ME1), which converts malate to pyruvate^19^.

Hyperglycaemic conditions in late stages of diabetic individuals include well known microvascular complications (nephropathy, neuropathy, retinopathy) and macrovascular complications (atherosclerosis and cardiovascular)^20–23^. Even these complications evidently are polygenic disorders^24–30^. Inflammation could lead to vascular calcification in association with atherosclerosis^31^. Hepatokines such as Fibroblast growth factor 21, Fetuin A and selenoprotein P are also implicated in the development of atherosclerosis^32^. It is evident that the complications arising from T2DM are also multifactorial and could be further governed by gene-environment interactions.

We have mined the voluminous literature available in the PubMed database for genes associated with T2DM and other complications. The available specific databases, namely, Type 2 diabetes Database (T2D-Db)^33^, T2DM Genetic Association Database (T2DGADB)^34^ and T-HOD^35^ although serving as useful resources by providing genetic association studies as well as integrated resources including gene expression, pathways and protein–protein interactions, however lack recent updates on the roles of specific genes identified as associated with complications in T2DM. We have supplemented our resource collection with data in these databases in a few cases. We believe, our up to date integrative data collection from genetic and functional gene expression studies would serve better for developing methods for risk assessment of T2DM associated complications and health forecasting.

## Results

**Figure 1:**
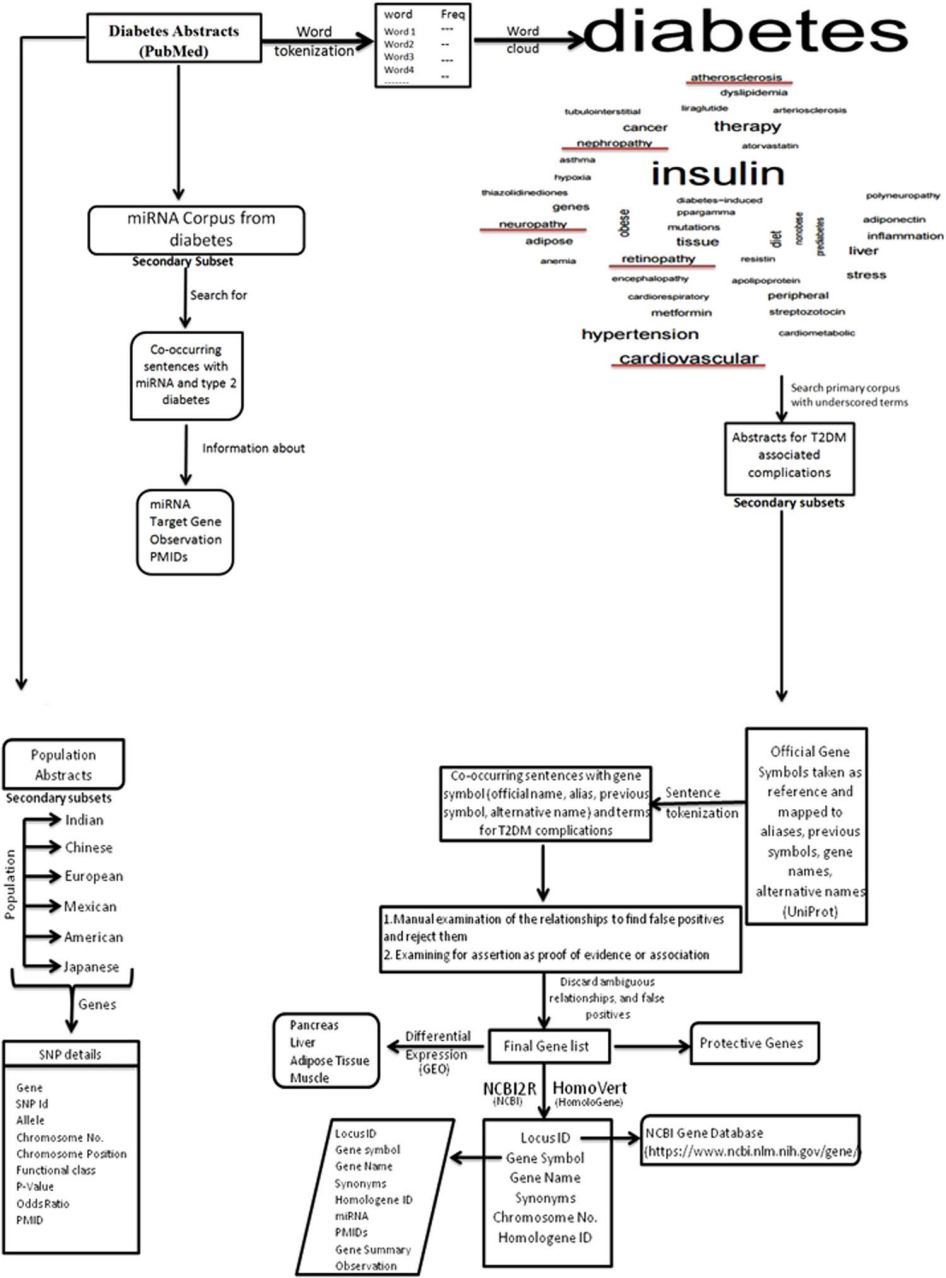
Work flow for text analytics of literature from PubMed. The data sources for each task are mentioned adjacent to the task name.

**Figure 2:**
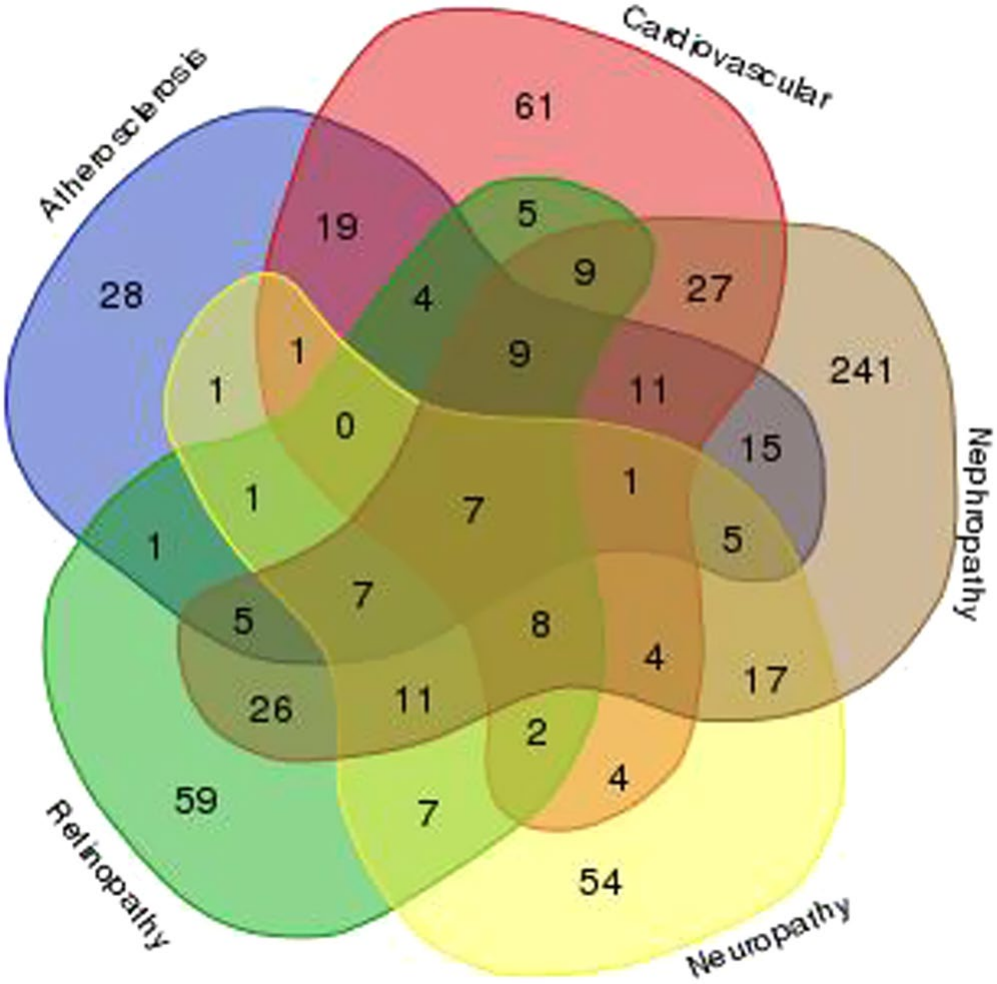
Venn diagram of genes implicated in T2DM complications. 7 genes encircled are *PON1, TNFRSF11B, CRP, NOS3, CRK, AGER, ADIPOQ* common in all complications.

**Figure 3:**
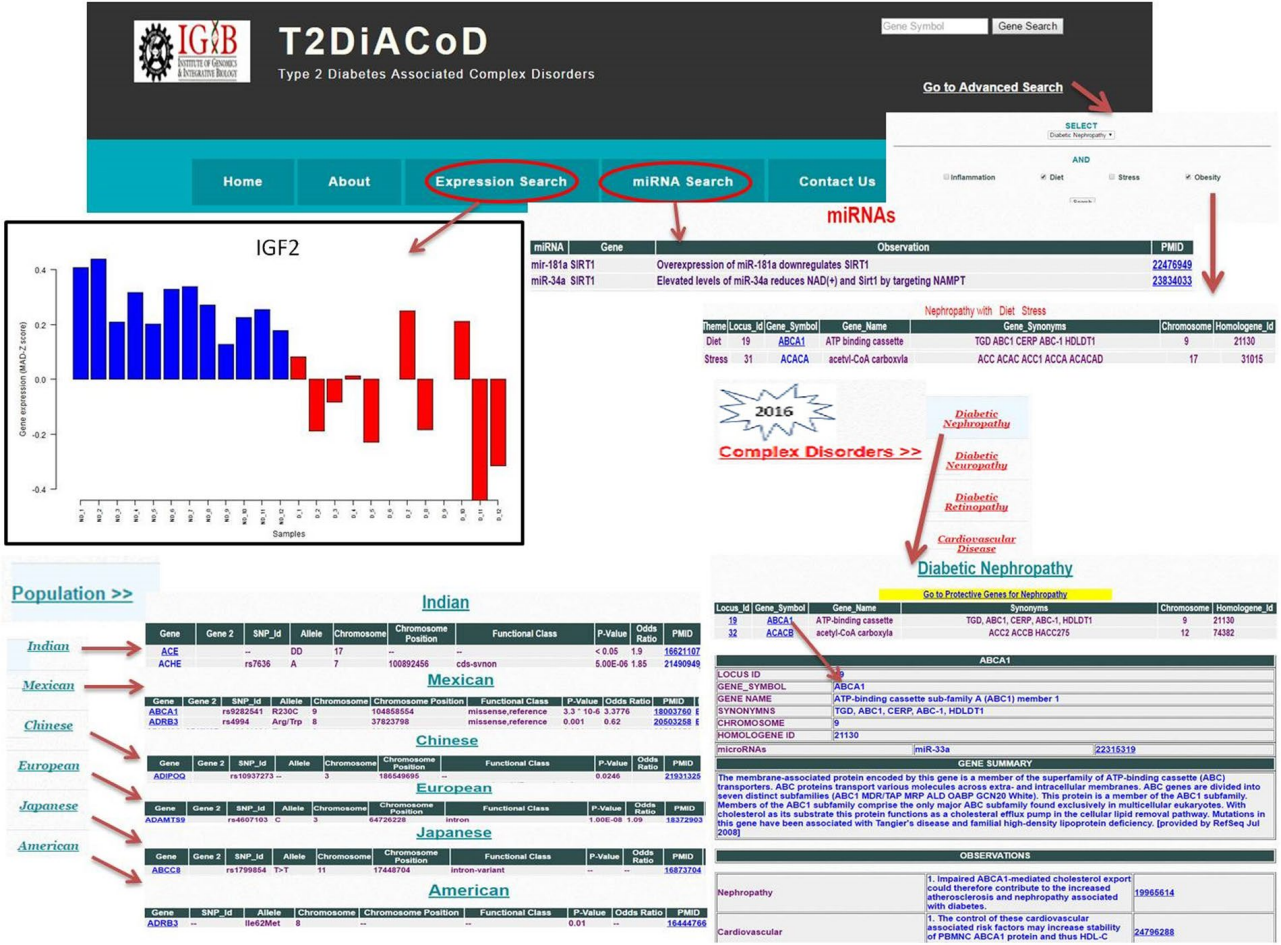
Visualization of T2DiACoD. The four sections of T2DiACoD are shown here as cropped screen shots. In T2DM complication, the nephropathy gene is displayed with gene summary (bottom right) and in the population section, example data for each of the populations in the database is shown. Histograms (top left) represent the differential expression of a gene *IL6*. miRNAs with their corresponding genes and observations are displayed top middle. An example of combination search is presented - nephropathy with diet and stress.

**Figure 4:**
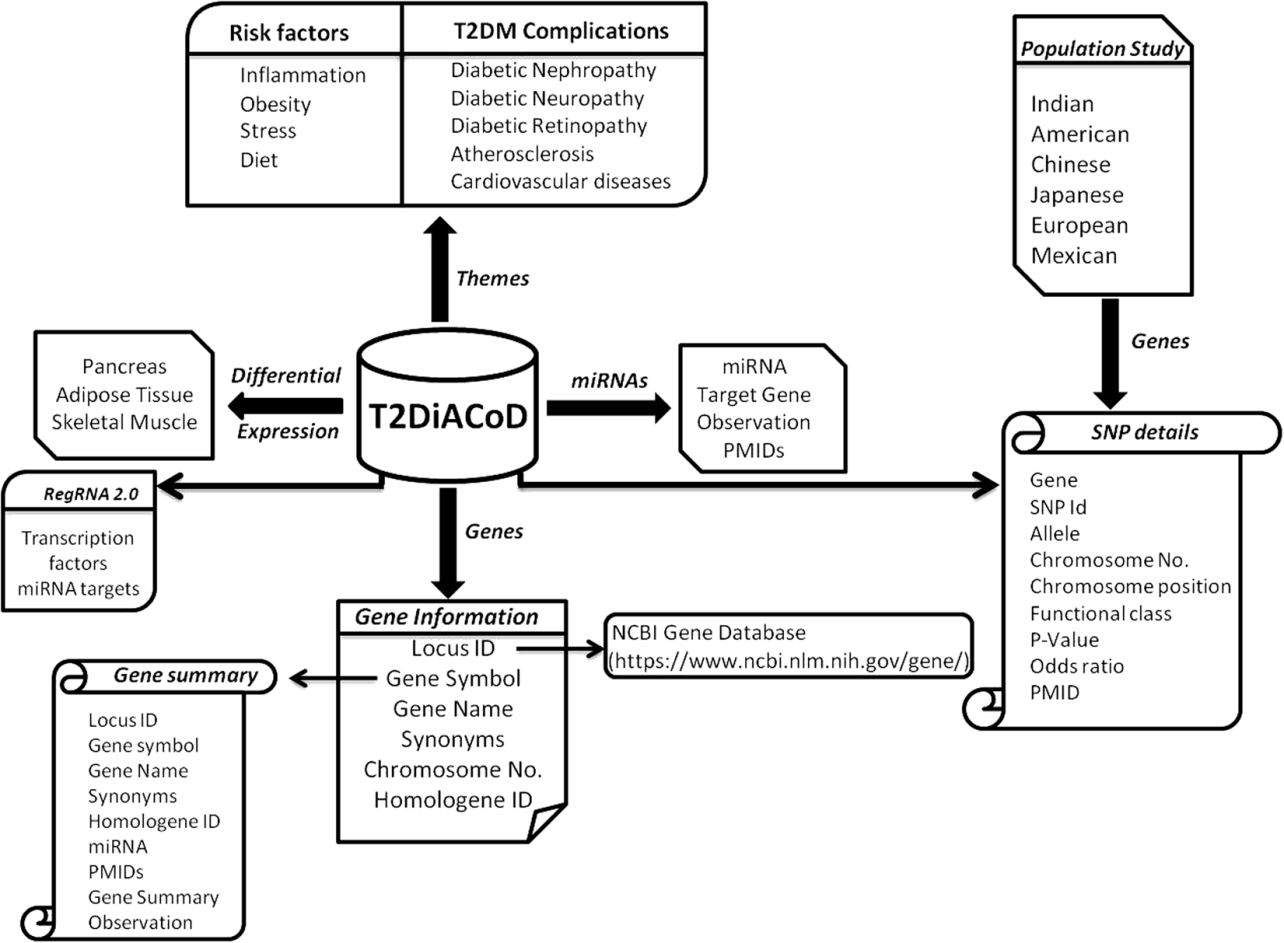
Database structure. T2DiACoD is a tetrapodic layout (i) T2DM associated complications, (ii) Gene expression (iii) miRNA for T2DM (iv) Population studies.

T2DiACoD contains a total of **650** non-redundant genes reported to be directly or indirectly associated with complications under diabetic conditions namely atherosclerosis, diabetic nephropathy, diabetic neuropathy, diabetic retinopathy, cardiovascular. The work flow for text analytics of literature from PubMed is shown in Fig. 1. Among the complications in T2DM condition, nephropathy (403 genes) tops the list followed by cardiovascular (172 genes), retinopathy (161 genes), neuropathy (130 genes), and atherosclerosis (115 genes). 34 microRNAs were associated with these complications and the miRNA hsa-miR-103/107 is common to atherosclerosis, nephropathy and neuropathy complications. Seven genes *AGER* (advanced glycosylation end product-specific receptor)*, TNFRSF11B* (tumor necrosis factor receptor superfamily, member 11b)*, CRK* (v-crk sarcoma virus CT10 oncogene homolog (avian))*, PON1* (paraoxonase 1)*, ADIPOQ* (adiponectin, C1Q and collagen domain containing), *CRP* (C-reactive protein, pentraxin-related) and *NOS3* (nitric oxide synthase 3 (endothelial cell)) are associated with all 5 complications (Fig. 2). The visualization of T2DiACoD is shown in Fig. 3 and structure of database is shown in Fig. 4.

### Comparison with other available resources

We have compared T2DiACoD from available resources, namely, DisGeNET^36^, T2D-Db^37^ and T2D Knowledge portal^38^. DisGeNET integrates the data on human-disease association from available databases and literature. Its data are categorized into 3 categories 1) Curated Data 2) Predicted Data and 3) Literature Data. DisGeNET has collected data from different sources i.e. UniProt, ClinVar, Orphanet, The GWAS catalog, CTD, RGD, MGD and literature data from GAD, LHGDN and BeFree. We observed that DisGeNET contains 705 genes, however several of these genes are either associated with Type 1 Diabetes or are false matches (either absence of strong evidence or presence of negative evidence in the text provided). T2D-Db provides information at molecular level for type 2 diabetes and its pathogenesis and there are 83 genes for T2DM associated complications, namely, nephropathy, neuropathy, retinopathy and cardiovascular diseases. T2D Knowledge portal is a database of DNA sequence, functional and epigenomic information, and clinical data from studies on type 2 diabetes and complications as well as provides analysis of this data. T2DiACoD containing 650 genes as on date is a repository, which stores information on genes associated with T2DM complications, protective genes and their roles, Drug targets, population SNPs data, expression analysis in different tissues of T2DM patients, miRNAs, 34 miRNAs and their target genes involved in associated complications of T2DM, interactions with risk factors, namely obesity, inflammation, diet and stress of genes associated with T2DM complications.

### Gene trends

We sought to identify the trends of genes studied in each complication by classifying them either as studied in a single year or are being studied in multiple years perhaps even to this day. These trends could be considered for new drug targets or markers for T2DM associated complications. Trends were computed using the Buzz Word Index (BWI). The results are displayed in Table 1 and the detailed results are presented in Supplementary Table 1. A few genes did not register a positive BWI value. Among the 23 drug targets (listed in T2DiACoD web site), 13 (*SGK1*, *SLC2A4*, *JAK2*, *HPSE*, *GCK*, *DPP4*, *CCR2*, *GIP*, *APOC3*, *PCSK9*, *PTPN1*, *SLC5A2*, *VCAM1*) have been studied in multiple years, three (*GLP1R*, *ERRFI1*, *LRP2*) have been studied in a single year. In case of markers or biomarkers, 2 genes each were studied in multiple (*CXCL16*, *RBP4*) as well as in a single year (*FGF21*, *HLA-DQA1*). Below, we describe the highlights of the results of trends of genes in each T2DM complication (Supplementary Table 6 for acronyms expansion).

**Table 1:** Number of Genes studied in a single year or in multiple years in T2DM complications.

	No. of Genes studied in Single Year only	No. of Genes studied in multiple Years
Diabetic Nephropathy	127	163
Diabetic Neuropathy	31	33
Diabetic Retinopathy	34	78
Cardiovascular Diseases	31	129
Atherosclerosis	28	59

#### Cardiovascular

In the case of cardiovascular disease, out of 172 genes there are 31 genes studied in a single year. The gene *ACVR1C* (activin A receptor type 1C) studied in 2015 with high BWI value 1547.5 is a member of *TGF-beta* receptors and is reportedly correlated in expression with pathogenic risk of T2DM as well as cardiovascular diseases. Silencing of *ACVR1C/ALK7* gene has reportedly protective effect on diabetes induced aortic stiffness, insulin resistance and hyperlipidemia^39^. 129 genes were studied in multiple years including *PON1, NOS3, TNF* (tumor necrosis factor)*, TNFRSF11B (OPG), HMGB1* (high mobility group box 1), and *ADIPOQ*. The gene *PON1* (paraoxonase 1), an anti-inflammatory high density lipoprotein (HDL)-associated enzyme, has been shown to decrease in diabetic patients^40, 41^, and its low concentration and enzymatic activity could be independent predictor of cardiovascular events in diabetic patients^42^. The synergic effect of *NOS3 (eNOS) Asp298* allele confirms its pathological role for cardiovascular diseases in T2DM patients^43^. Blazquez-Medela *et al*. (2012) studied relationship between serum TNFRSF11B (Osteoprotegerin (OPG)) and vascular alteration in associated pathologies and its levels were found to be higher in diabetic, hypertensive with retinopathy and cardiovascular risk patients and therefore associated with cardiovascular risk in diabetes and hypertension patients^44^. The G allele of *ADIPOQ* (adiponectin) G276T polymorphism is a susceptible allele for cardiovascular disease in T2DM patients^45^ and increased serum HMGB1 level was associated with cardiovascular diseases in T2DM patients^46^.

#### Atherosclerosis

28 Genes were studied in a single year in atherosclerosis. *DHCR7* (7-dehydrocholesterol reductase) with BWI 135.8 and *B2M* (beta-2-microglobulin) with BWI 359.8 had high BWI values indicating good emphasis of the corresponding genes in these studies. *DHCR7* was recently investigated for its role in T2DM to influence subclinical atherosclerosis using Mendelian randomization approach and carotid artery intima-media thickness (cIMT) measurements^47^. Strawbridge *et al*. observed *rs3829251* (*DHCR7*) influenced the progression of atherosclerosis in T2DM patients. Glycation of *B2M* may contribute to the risk of diabetes associated complications. Kim *et al*. in 2014 reported higher serum B2M as an independent risk factor for atherosclerosis and diabetic nephropathy (DN) in T2DM patients^48^. Genes *IRAK4, HDAC9, ACSL1 and APPL159* are recently reported as atherosclerosis causing genes in T2DM patients. 59 genes were reportedly being investigated in multiple years for their role in atherosclerosis, for example the genes *ABCA1, PON1, MTHFR, PLTP, PPAR, TNFRSF11B (OPG), FABP4*, and *IL18* are being studied for several years since 1997 with varying emphasis. *ABCA1* (ATP-binding cassette transporter A1) was noted as atheroprotective protein^49^ in that it protects against the formation of atherosclerosis and macrophage ABCA1 protects arteries from promoting atherosclerosis lesions^50^. Polymorphism or genetic aberrations in *ABCA1* gene can be associated with severity of atherosclerosis^51^. Sartippour *et al*. (2000) evaluated the *in vitro* and *ex vivo* effect of high glucose concentrations on macrophage *PPAR* (Peroxisome proliferator-activated receptor) mRNA expression^52^ and observed that dysregulation of macrophage *PPAR* expression in T2DM alters arterial lipid metabolism and inflammatory response and might contribute to the accelerated atherosclerosis in T2DM. Upregulation of *FABP4* (fatty acid binding protein 4) further enhances the macrophage lipid accumulation by advanced glycation end products (AGE), which further accelerates formation of foam cells and development of atherosclerosis in diabetic patients^53^. Nakamura *et al*. studied whether serum level of IL-18 (Interleukin 18) is a common predictor of nephropathy and atherosclerosis in T2DM patients. They observed that serum and urinary level of IL-18 were significantly elevated in T2DM patients compared to controls thereby indicating that serum levels of IL-18 could be a predictor for these complications^54^.

#### Nephropathy

Diabetic nephropathy is a widely studied complication and the list of genes reportedly involved in the cause or progression of diabetic nephropathy is larger than in other complications. There are 127 genes out of 403 studied in a single year. A few examples are, *CHN2, CNR, GAS1, IL1B, IRS2, PFKFB2, RREB1*, and *TRIB3*. Genes *LRP2, NGAL, IGFBP7, CUBN, CHIT1* are recently reported genes in diabetic nephropathy. The *TRIB3* ‘G’ allele was identified as associated with diabetic nephropathy and it was suggested that this observation may help to improve targeting of therapy to diabetic patients^55^. Zhang *et al*. characterized diabetic nephropathy (DN) by mesangial proliferation and glomerular hypertrophy. microRNAs are implicated in this pathogenesis. They discovered hsa-miR-34a regulated mesangial proliferation and glomerular hypertophy by directly inhibiting *GAS1* in early DN^56^. In case of *IL1B* the C511T variant is reported associated with development of DN^57^. IRS2 is discussed as marker or mediator of human DN^58^. *RREB1* is considered as novel candidate gene for T2DM associated kidney diseases^59^. 163 genes out of 403 are studied in multiple years. The genes for example, *AGT, CTGF, EPO* and *MTHFR* were observed as being investigated with importance. The C677T mutation in *MTHFR* (Methylenetetrahydrofolate reductase) gene was reported as risk factor for DN in 1999 by Shcherbak *et al*.^60^ and Mazza *et al*.^61^ and was highly studied gene in 1999 with BWI 436.8.

#### Neuropathy

In diabetic neuropathy, 31 genes were studied in a single year, for example, *HMGB1, IGFBP5* and *SERPINF1 (PEDF)*. Abu El Asrar *et al*. (2014) hypothesized that increased expression of *HMGB1* (high mobility group box-1) gene, a proinflammatory cytokine, is responsible for pathogenic role in mediating diabetes induced retinal neuropathy and observed that early retinal neuropathy of diabetes involves up-regulated expression of *HMGB1* and can be mitigated by inhibition of *HMGB1* ^62^. Simon *et al*. (2015) observed that elevated expression of *IGFBP5* in diabetic nerves of mice leads to the progressive neurodegeneration and could offer novel treatment strategies for diabetic neuropathy (DNP)^63^.

The number of genes studied in multiple years are 33 including *ACE* and *NGF*. Hellweg *et al*. (1990) suggested that NGF is required for the development and maintenance of peripheral neurons, therefore changes in their levels could underlie diabetic neuropathy^64^. Fradji *et al*. (2013) measured the serum levels of NGF and found lower level of NGF in patients’ with diabetic neuropathy^65^. Recently in 2014 it was observed that Vitamin A increases the level of NGF, which helps in improving diabetic neuropathy in rats^66^.

#### Retinopathy

In diabetic retinopathy 34 genes including *AP15, GRB2, IL17A, PLXDC1, C5* and *L1CAM* were studied in a single year in which, *C5* and *L1CAM* are reported as recently as in 2016. For example, Burdon *et al*. (2015) reported genetic variation on chromosome 17q25.1 near *GRB2* as associated with diabetic retinopathy and expression of *GRB2* is up-regulated during retinal stress and neovascularisation^67^. Yamaji *et al*. (2008) determined whether TEM7 (PLXDC1) is associated with formation of fibrovascular membranes (FVMs) further responsible to cause proliferative diabetic retinopathy (PDR) and observed significant role of TEM7 in the proliferation and maintenance of neovascular endothelial cells in FVMs and also could be molecular target for new diagnostic of PDR^68^.

Nearly 48.44% of genes (78 genes) were studied in multiple years including *ACE, AGER, ADIPOQ, SERPINF1* and *SIRT1*. Pradeepa *et al*. (2015) recently proposed to assess the association of ADIPOQ (adiponectin) and microvascular complications of diabetes and observed that serum adiponectin levels were associated with diabetes associated complications and also with severity of retinopathy^69^. Niu *et al.* (2012) provided convincing evidence that *RAGE (AGER)* gene 1704T allele is associated with increased risk of diabetic retinopathy^70^.

### Gene enrichment analysis

The numbers of genes annotated with few gene ontologies (GOs) are far greater than that annotated with a large number of GOs in all 5 complications.

The GOs unique to cardiovascular complications are: circulatory system process, blood circulation, lipid localization, lipid transport, lipid binding. The cardiovascular complications arise due to misbalanced lipid levels in the blood and are considered to be responsible to affect blood vessels; Coronary heart disease (CHD) occurs when arteries supplying blood to the heart muscle becomes blocked. Therefore, blood circulation and lipid transport significantly affect cardiovascular functions. The genes *ADIPOQ, ACE2, APOE* and *CETP* are implicated in these processes and polymorphism in these genes have been reported responsible to cause cardiovascular diseases (CVD) in diabetic patients^71–74^. The unique GOs found for genes implicated in cardiovascular disease are displayed in Supplementary Table 2a.

**Figure 5:**
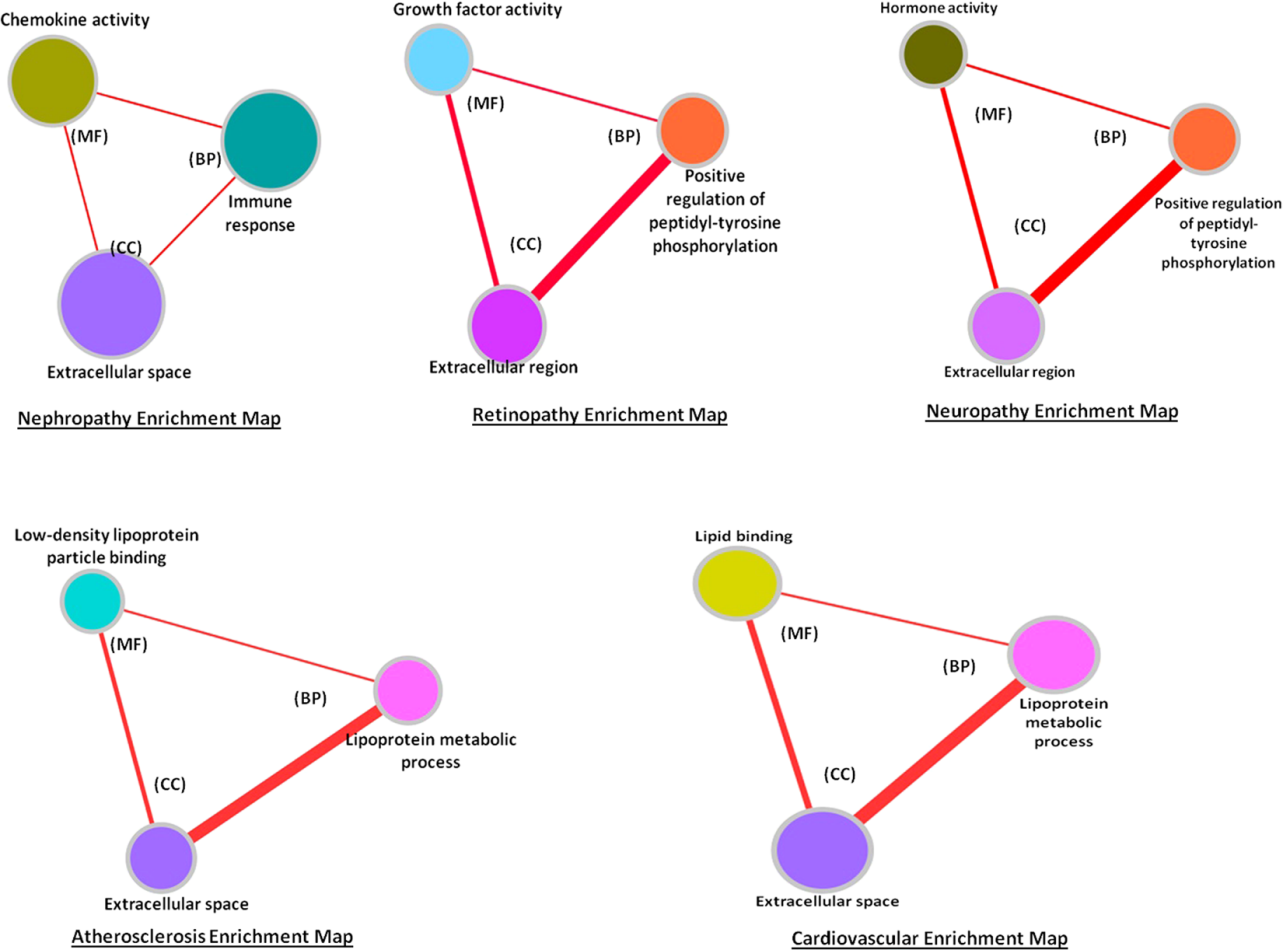
Gene-set enrichment visualization using EnrichmentMap. In the graph, nodes represent gene set involved in the GO term mentioned adjacent to the node and edges represent the extent of overlap of genes between the two gene sets connected by them. Node size and edge width are proportional to the number of genes. Colour representation Purple = Extracellular space, Violet = Extracellular region, Orange = Positive regulation of peptidyl-tyrosine phophorylation, Olive = Chemokine activity, Army green = Hormone activity, Pine green = Immune response, Light blue = Growth factor activity, Turquoise = Low-density lipoprotein particle binding, Yellow green = Lipid binding, Pink = Lipoprotein metabolic process.

In the case of atherosclerosis 27 unique GOs were observed, namely, lipid rich processes, cholestrol homestasis, lipoprotien metabolc process, high density particle process. Ait-Oufella *et al*. (2011)^75^ reviewed the role of cytokines in the cause of atherosclerosis and observed that blockage of proinflammatory cytokines could limit the plaque development and progression^75^. Genes implicated in these GOs are for example *IL6, TNF, TLR4* and *ADIPOQ*. It is reported that homocysteine upregulates Matrix metalloproteinases-tissue inhibitor of metalloproteinases (MMP-TIMP) pathway and *IL6* release, the effect being stronger in the presence of high glucose, which further contributes to increased atherogenesis in diabetic patients^76^. Advanced glycation end-product of low-density-lipoprotein (AGE-LDL) activates TLR4-mediated signaling pathway, thus inducing proinflammatory cytokine production, with increased risk of atherosclerosis in diabetics^77^. The unique GOs found for genes implicated in atherosclerosis are displayed in Supplementary Table 2b. The GO enrichment map displayed in Fig. 5 shows the high similarity between the two maps of atherosclerosis and cardiovascular.

In micro-vascular complications, large numbers of unique GOs are enriched, for e.g. 47 in nephropathy, 25 in retinopathy and 20 in neuropathy. Diabetic nephropathy (DN) is accompanied by renal failure^78, 79^ and is associated with cardiovascular complications^80^. Ni *et al*. discussed the various signaling pathways with respect to development, progression and prevention of hyperglycaemia induced diabetic nephropathy^81^. It is notable that the number of genes implicated in DN far exceeds than that in other diabetic complications. Consequently, we observed higher number of unique GOs (41) associated with the genes implicated in DN. Regulatory processes, signaling, kinase, redox coupling encompasses diabetic nephropathy. the genes in MAPK and JAK-STAT pathways are associated with these GOs and involved in the pathogenesis of DN^82^. Overall, the GO terms unique for the genes implicated in DN explain its pathophysiology and therefore these genes could specifically be investigated for their potential for marker. The unique GOs found for genes implicated in diabetic nephropathy are displayed in Supplementary Table 2c.

Diabetic retinopathy is the result of damaged blood vessels supplying blood to retina due to high sugar level. The unique GOs dominant in regulation. Genes such as *CTGF, VEGFA, VEGFC* and *TCF7L2* are implicated in these processes. *TCF7L2* promotes pathological retinal neovascularization via ER stress-dependent up regulation of *VEGFA* ^83^. CTGF (connective tissue growth factor), downstream effectors of angiogenesis in diabetic retina, could be possible target for therapeutic application of diabetic retinopathy^84^. Kaidonis *et al*. (2015) investigated the association between SNP in *VEGFC* and diabetic retinopathy (DR) in T1DM and T2DM patients’ and observed significant associations, namely, three *VEGFC* SNPs associated with DR: rs17697419, rs17697515 and rs2333426^85^. In addition we found *ALDH1A1, RBP4, APOB, APOA1, RBP1, APOE* and *RHO* genes enriched in retinoid metabolic process as unique GO in diabetic retinopathy. The unique GOs found for genes implicated in diabetic retinopathy are displayed in Supplementary Table 2d.

Diabetic neuropathy is associated with capillary dysfunction^86^, which is clinically manifested by vascular and metabolic alterations^87, 88^. The unique GOs encompass hormone activity, regulation and immune response. Tsuzuki *et al*. (1998) determined the influence of *APOE* phenotype in the progression of peripheral neuropathy in diabetics^89^. The decreased expression of *SOD2* (superoxide dismutase) increases the risk of diabetic neuropathy^90^. *PDE5* (phosphodiesterase-5) is upregulated in diabetic condition, its inhibitor sildenafil activates cGMP/PKG signaling pathway and mediates beneficial effect on diabetic neuropathy^91^. The unique GOs found for genes implicated in diabetic neuropathy are displayed in Supplementary Table 2e. The GO enrichment map displayed in Fig. 5 shows the distinctness of nephropathy map but high similarity between the two maps of neuropathy and retinopathy.

### miRNAs

34 miRNAs and their target genes involved in associated complications of T2DM are shown in Supplementary Table 3. The target for hsa-miR-103/107 common to atherosclerosis, nephropathy and neuropathy complications is *CAV1* involved in viral myocarditis, Endocytosis, Proteoglycans in cancer, Focal adhesion and Bacterial invasion of epithelial cells pathway.

Significant accumulation of malonyl CoA accompanied by ER stress induction is mediated by over-expression of miR-107. Increase in levels of miR-107 is critical and promotes lipid accumulation in hepatocytes and this might form the basis of diverse etiologies encountered in a fatty liver. Lipid accumulation is induced by miR-107 and this is mediated by endoplasmic reticulum (ER) stress. The ER stress inhibitor, 4-Phenyl butyric acid (4-PBA) significantly decreased such miR-107 induced lipid accumulation. *db/db* mice are well-known genetic models for NAFLD. hsa-miR-107 levels that are elevated in the *db/db* mice liver, induce ER stress and promote lipid accumulation in liver cells by targeting Fatty acid synthase (FASN)^92^.

Two members of hsa-miR-33 family called mir-33a and mir-33b, are located in intronic regions within two protein-coding genes for Sterol regulatory element-binding proteins (SREBP-2 and SREBP-1) respectively. It has been shown that adenoviral hsa-miR-33a overexpression in human or mouse islets reduced *ABCA1* expression, decreased glucose-stimulated insulin secretion, and increased cholesterol levels. Therefore, hsa-miR-33a regulates *ABCA1* expression in pancreatic islets, thus affecting cholesterol accumulation and insulin secretion^93^.

Two miRNAs hsa-miR-192 and hsa-miR-193b are increased significantly in the pre-diabetic state. Strikingly, in plasma of glucose-intolerant mice these miRNAs are also increased. After a therapeutic intervention consisting of chronic exercise, the circulating levels of hsa-miR-192 and hsa-miR-193b returned to baseline in both pre-diabetic humans and glucose-intolerant mice thereby succeeding in normalizing metabolic parameters^94^.

Hsa-miR-194 is under regulation by *HNF1A* and a higher expression level is found in liver and intestinal epithelial cells. *HNF1α* is important for proper functioning of β-cell and mutations in this gene causes maturity onset diabetes of the young (MODY)^95^.

Obesity-induced overexpression of hsa-miR-802 impairs glucose metabolism through silencing of *HNF1B* ^96^. The levels of circulating hsa-miR-101, hsa-miR-375 and hsa-miR-802 are significantly increased in T2DM patients versus non-glucose tolerance (NGT) subjects and they may become the new biomarkers for type 2 diabetes^97^.

microRNA 21 encoded by the *MIR21* gene was one of the first mammalian microRNAs identified. The dynamic biomarkers for systemic inflammatory or angiogenic status are circulating miR-21-5p and miR-126-3p. The expression levels in circulating angiogenic cells (CACs) from T2DM with major cardiovascular events (MACE) suggest a shift from a proangiogenic to a proinflammatory profile^98^. The hsa-miR-21 modulates the PTEN-AKT pathway and thus antagonises the insulin resistance in adipocytes. The hsa-miRNA-21can be a new therapeutic target for metabolic disease such as T2DM and obesity^99^.

The hsa-miR-181 microRNA precursor is a small non-coding RNA molecule regulating *SIRT1* and improves hepatic insulin sensitivity. Inhibition of miR-181a might be a potential new strategy for treating insulin resistance and T2DM^100^.

The level of beta-cell apoptosis increases with small changes in hsa-mir-34a. Further studies can be carried out for determining the effect of rare variants on type 2 diabetes^101^.

Encoded by the *MIR155* host gene, hsa-miR-155 in humans plays an important role in various physiological and pathological processes. Exogenous molecular control *in vivo* of miR-155 expression may inhibit malignant growth, viral infections, and attenuate the progression of cardiovascular diseases. Down regulated levels of hsa-miR-155 could play an important role in the pathogenesis of T2DM due to their relationship with metabolic control^102^. Hsa-miR-155 has a direct target *NR1H3* (LXRα), which is potentially responsible for liver phenotype of miR-155(−/−) mice. This micro RNA also plays a crucial role in regulation of lipid metabolism as its dysregulation might lead to hepatic steatosis in diabetic patients^103^.

Silencing of the most abundant miRNA, hsa-miR-124a expressed in neuronal cells in T2DM islets results in increased expression of target genes important for beta cell function. Simultaneously, overexpression of these genes stimulates insulin secretion indicating that expression of miR-124a might contribute to beta cell dysfunction in T2DM^104^.

Transcription of a “common miRNA signature”, hsa-miR-144 precursor is activated by GATA4. Signature miRNAs, that could possibly explain the pathogenesis of T2DM and the significance of the miRNA in insulin signalling^105^.

Insulin receptor Substrate 1 (*IRS1*) a target for hsa-miR-144 and is involved in insulin signaling at mRNA and protein level is also directly down-regulated with increased expression of the miRNA. Plasma miR-144 is increased in patients with T2DM and has also been reported to be involved in regulation of insulin sensitivity in muscle tissue^106^.

Peroxisome proliferator-activated receptor gamma co activator (PGC)-1α and estrogen-related receptor (ERR)-α expression were upregulated by both overexpression and downregulation of hsa-mir-106b. hsa-miR-106b targets *MFN2* and regulates skeletal muscle mitochondrial function and insulin sensitivity. This could offer a potential for new strategy for treating insulin resistance and sensitivity and T2DM^107^. Brown adipose tissues of high fat diet-induced mice have higher expression levels of hsa-miR-106b and hsa-miR-93. Moreover, the mRNA level of *UCP1* was suppressed by the expression of the two miRNAs, hsa- miR-106b and miR-93^108^.

The two miRNAs (hsa-miR-25 and hsa-miR-92a) could control insulin synthesis directly by targeting the 3′UTR of insulin gene (*INS*). Furthermore, introduction of anti-miR-25 or hsa- miR-92a leads to increased insulin synthesis, which was suppressed by miR-9^109^.

MicroRNA (miRNA)-463-3p direct targets *ABCG4* inhibits Glucose-stimulated insulin secretion (GSIS) essential for control of metabolic fuel homeostasis^110^. In type 2 diabetes human pancreatic islets, as compared with non-diabetic controls, up - regulation of insulin and expression of miRNA-463-3p and down-regulation of ABCG4 were observed and their expression levels were closely related.

miR-194 involved in PI3K-Akt signaling pathway was significantly reduced by 25% to 50% in both rat model and human with pre-diabetes and established diabetes^111^. hsa-miRNA-194 targets *Akt1*. Interestingly, hsa-miR-194 was a unique miRNA that appeared regulated across different stages of the disease progression from the early stages of insulin resistance to the development of T2DM^111^.

hsa-miRNA-15b having targets *INSR* and *CCND1* was disregulated by diet-induced obesity (DIO) or palmitate in hepatocytes^112^. Mice with diet-induced Obesity developed hyperglycemia and insulin resistance associated with a reduction in expression of Insulin receptor (*INSR*). In addition, protein expression of *INSR* was suppressed by the over expression of hsa-miR-15b through targeting INSR 3′ untranslated region directly^112^. This resulted in an impairment of insulin signaling and glycogenesis in hepatocytes. A causal link of miR-15b has been reported to the pathogenesis of hepatic insulin resistance in SFA-induced obesity. *INSR* gene is involved in 12 pathways of which, Type II diabetes mellitus, PI3K-Akt signaling pathway, Insulin signaling pathway (FDR < 0.05) are prominent related to Type 2 Diabetes whereas *CCND1* is involved in Jak-STAT signalling pathway apart from PI3K-Akt signaling pathway^112^.

A significant increase of hsa-miR-10a was observed with the knockdown of *HDAC3* with siRNA, resulting in decrease of *CREB1* the target of miR-10a, and fibronectin (FN) expression in kidney of HFD/STZ mice^113^. On the contrary, over expression of *HDAC3* decreased miR-10a content, enhanced albumin-to-creatinine (ACR) value, *CREB1* and FN in mice. To sum up,it has been elucidated that *HDAC3*/miR-10a/*CREB1* serves a new method underlying kidney injury,providing potential therapeutic target in type 2 diabetes^113^.

### Protective Genes

During the search for genes responsible for T2DM associated complications we observed some genes could offer protection from the disease either by their up regulation or down regulation or genetic association. The numbers of genes offering protective effect were 33 in nephropathy, 14 in neuropathy, 13 in retinopathy, 11 in cardiovascular diseases and 4 in atherosclerosis. We observed 19 of these genes differentially expressed in T2DM patients with *Padj* <=0.05, namely, *ADCYAP1, ALDH2, APPL1, CAT, CDKAL1, DCXR, ERBB3, FGF21, HDAC4, HLA-DQA1, NFE2L2, NGF, PECAM1, PROC, RBP4, SIRT6, SYVN1, TIMMP4* and *TRPV4*. None of the genes had fold change (FC) either >2 or <0.5. Example genes with function and mechanism of protection against respective complications are described below.

A few genes *ACE2*, *ADCYAP1*, *NCF1*, *NFE2L2*, *OSM*, *SMAD1*, *TGFB1*, *BDNF*, *SYVN1*, *TXNIP*, *CD36*, *CYP2J2* and *NLRP3* are described below with their function and protective role in corresponding diseases.

#### Cardiovascular

The *NLRP3* and *CYP2J2* provide protection against cardiovascular (CVD) complications in T2DM patients. NLRP3, NLR family, pyrin domain containing 3, is a member of NALP3 inflammasome complex. Silencing the *NLPR3* gene might protect against diabetic cardiomyopathy^114^. *CYP2J2*, cytochrome P450, family 2, subfamily J, polypeptide 2, is a member of cytochrome P450 superfamily of enzymes and is responsible for epoxidation of endogenous arachidonic acid in cardiac tissues. Cardiac specific overexpression of *CYP2J2* protect against diabetic cardiomyopathy^115^.

#### Atherosclerosis

CD36 is a lipid and fatty acid receptor that plays an important role in the metabolic syndrome and associated cardiac events^116^. In rodent models Geleon A *et al*. show that CD36 inhibitors reduce postprandial hypertriglyceridemia and protect against diabetic dyslipidemia and atherosclerosis.

The gene *NFE2L2* contributes in the protection against all 3 micro-vascular complications. *ACE2* and *ADCYP1* offer protection against diabetic retinopathy as well as diabetic nephropathy. *NCF1, OSM, SMAD1* and *TGFB1* provide protection against diabetic nephropathy, *SYVM1* and *TXNIP* protect against diabetic retinopathy and *BDNF* in ameliorating the diabetic neuropathy.

The gene *NFE2L2*, Nuclear factor, Erythroid 2 Like 2, is a transcription factor, regulating oxidative stress and also has an anti-inflammatory effect. In nephropathy, activation of *NFE2L2* reduces oxidative damage and negatively regulates *TGFB1* & extracellular matrix production^117^. In retinopathy, Xu Z *et al*. has described the protective role of *NFE2L2* in retina. *NFE2L2* regulates antioxidant genes via binding of ARE (Antioxidant Response Elements) and NFE2L2/ARE dependent signalling can cancel out diabetic retinopathy mediated injuries in retinal neurons^118^. In Neuropathy the expression of *NFE2L2* & *HMOX1* is down-regulated in sciatic nerves of diabetic mice and its expression aids in reduction of formalin induced inflammatory pain and thereby indicating its role in preventing sensory motor alterations^118^. Negi *et al*. found that decrease in NF-κB activation cascade and oxidative stress by increasing NFE2L2 may offer neuro-protective effect in diabetic neuropathy^119^.

*ACE2*, angiotensin 1 converting enzyme 2, functions in the regulation of cardiovascular and renal functions. Its expression is gradually decreased resulting in accumulation of ang11 in kidney leading to renal injury^120^. Increased expression of *ACE2* overcomes the impaired balance of retinal RAS and confers protection against DR^121^. *ADCYAP1*, adenylate cyclase activating polypeptide 1, stimulates adenylate cyclase and cyclic adenosine monophosphate (cAMP) levels. *ADCYAP1* provides protection in diabetic retina by attenuating neuronal cell loss in DR and mediating through the activation of PAC-1 receptor^122^. Its anti-inflammatory, anti-apoptotic and anti-fibrotic properties could aid in ameliorating DN.

#### Nephropathy

*NFE2L2*, nuclear factor, erythroid 2, is a multicomponent enzyme activated to produce superoxide anions. It provides protection against DN through the inhibition of TGF-beta1 and reduction in the production of extracellular matrix^123^. *OSM*, oncostatin M, is a member of cytokine family and regulates production of cytokines IL-6, G-CSF and GM-CSF. Tubular epithelial cell-myofibroblast transdifferentiation (TEMT) induced by OSM by activating JAK/STAT pathway could be inhibited by SOCS. Liu *et al*. reported that SOCS proteins inhibit OSM as well as TEMT induction and has a therapeutic effect in DN^124^. *SMAD1*, SMAD family member 1, is a signal transducer and transcriptional modulator. Decrease in SMAD1 and collagen type IV provides protection in DN^22^. *TGFB1*, transforming growth factor, beta 1, is a multifunctional peptide that regulates proliferation, differentiation and adhesion. Increased TGFB1 increases the production of extracellular matrix space (ECM) through activation of NOX4. Reduction in NOX4/TGFβ-1 signaling may provide therapeutic potential against DN^125^.

#### Neuropathy

In neuropathy, *BDNF*, brain derived neurotrophic factor, is a member if nerve growth factor family. It is necessary for the survival of striatal neurons in the brain. Increased BDNF likely contributes to reduction in Kv channel function through TrkB receptor stimulation with potential therapeutic effects in diabetic neuropathy^126^.

#### Retinopathy

In retinopathy, *SYVN1*, syniviolin 1, is involved in endoplasmic reticulum associated degradation. SYVN1 confers diabetic retinopathy resistance. Yang S *et al*. described this with expression analysis and found lower expression of *SYVN1* in diabetic mice^127^. *TXNIP*, thioredoxin interacting protein, is a thioloxidoreductase. It protects cells from oxidative stress. TXNIP plays crucial role in the inflammation and retinal injuries in early stages of DR^128^.

### Drug Targets

We have collected data on 23 recently identified drug targets for T2DM and its associated complications. 16 drug targets are reported for T2DM only and 7 others are for associated complications. For e.g. increased expression of solute carrier family 2, member 4 (*SLC2A4*) is beneficial for the treatment of insulin resistance^129^. Only *JAK2* was differentially expressed and down regulated in T2DM patients’ adipose tissue. *JAK2* is associated with diabetic nephropathy. *JAK2* is recognized target for T2DM targeting JAK-STAT pathway^130^.

### Differentially expressed genes from population studies

The genes collected from GWAS study in different populations namely Indian, American, Chinese, Japanese, European and Mexican, were examined for their differential expression in T2DM patients. These differentially expressed genes could possibly underlie fundamental biological processes perhaps beyond genetic differences between individuals. Out of 500 genes reported from all populations where 249 are unique genes, we obtained 29 differentially expressed genes and 2 genes had FC either >2 or <0.5. These genes are *IRAK1* and *VEGFA. VEGFA* is up regulated (FC = 7.91) in skeletal muscle tissues of T2DM patients. *VEGFA* is associated with 4 complications: nephropathy, neuropathy, retinopathy and atherosclerosis^131–133^. *IRAK1* is down regulated (FC = 0.008) in skeletal muscle tissues of T2DM patients and it is associated with neuropathy.

### Differential expression, multiple tissue involvement, presence in plasma and risk factors

We analysed individually 11 datasets published on T2DM patients’ tissues pancreas, adipose, skeletal muscle and liver. We have considered differentially expressed genes in different tissues in any of the microarrays, because there could be multiple pathways leading to the disease. We obtained a total of 227 differentially expressed T2DiACoD genes. 191 genes were differentially expressed in pancreas, 34 genes were differentially expressed in adipose tissue and 21 genes were differentially expressed in skeletal muscle. As per the criteria of Padj < 0.05 none of the T2DiACoD genes were significantly differentially expressed in patients’ liver tissues. 20 genes had FC values either >2 or <0.5. Of these, 20 genes, 1 is in adipose tissue and 19 are in skeletal muscle. We sought information on the sub-cellular location of the proteins encoded by the differentially expressed genes. Among those that were annotated as secreted, we further interrogated for their presence in plasma through text mining. In the following, only the differentially expressed genes at Padj < 0.05 are presented.

#### Cardiovascular

Among the genes implicated in cardiovascular complications, there are 56 genes differentially expressed in T2DM patients. These include 49 genes in pancreas, 8 genes in adipose tissue and 4 genes in skeletal muscle. Genes *FABP5, SLC2A1* and *TXN* were differentially expressed in pancreas and adipose tissue whereas *CD9* and *TXN* were differentially expressed in Pancreas and skeletal muscle. Seven differentially expressed genes *CST3, FGF21, IL18, INS*, and *RBP4* are annotated as secreted and their product proteins are reportedly present in plasma^134–142^.

#### Atherosclerosis

Among the genes implicated in atherosclerotic complications, there are 37 genes differentially expressed in T2DM patients. These include 28 genes in pancreas, 8 genes in adipose tissue and 4 genes in skeletal muscle. Genes *TXN* and *FABP5* were differentially expressed in adipose and pancreas. *TXN* was differentially expressed in all pancreas, adipose and skeletal muscle. Nine differentially expressed genes *FGF21, IL18, INS, LEP, RBP4, ANGPTL2, MIF* and *VEGFA* are annotated as secreted and are reportedly present in plasma^136–140, 143–145^. Two of these genes *ANGPTL2* and *VEGFA* had FC values either >2 or <0.5.

#### Nephropathy

**Figure 6:**
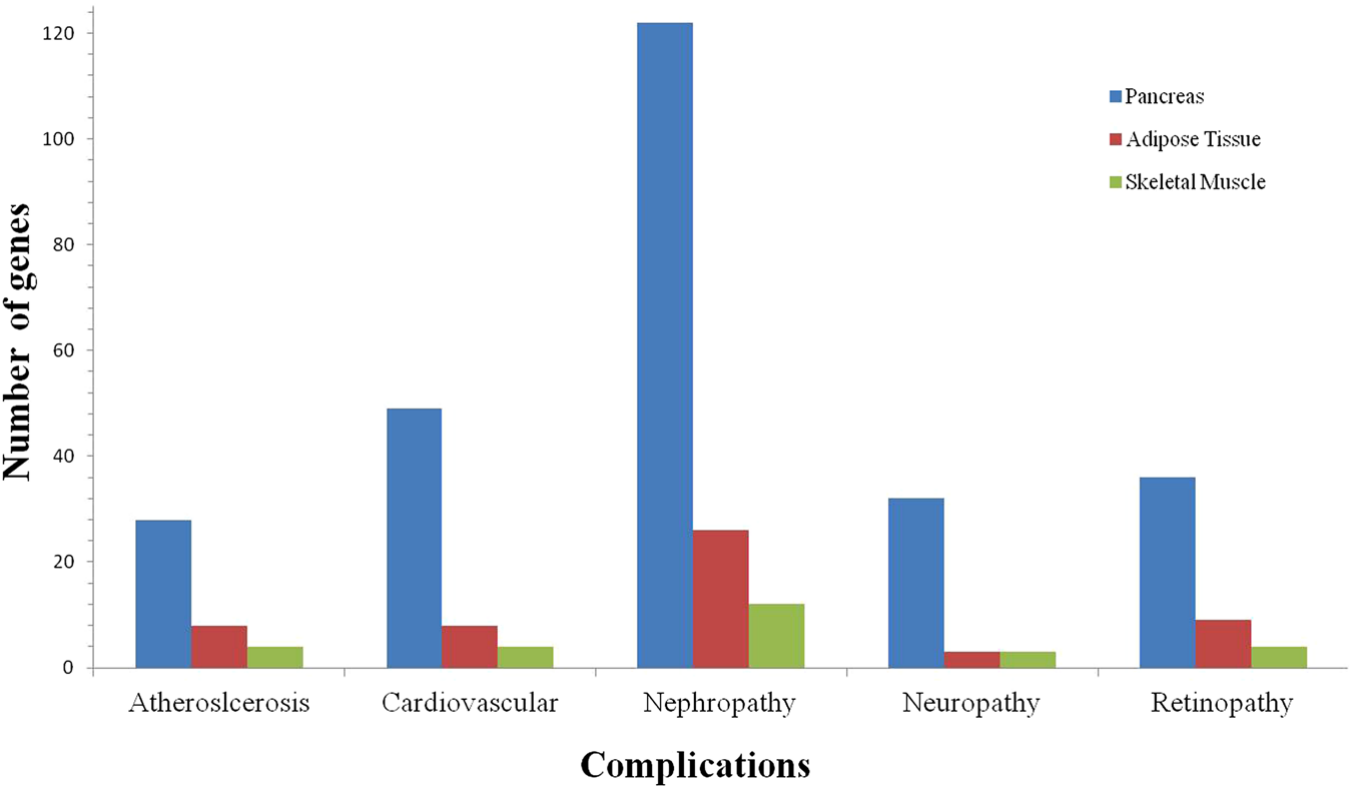
Number of differentially expressed genes with *padj* < =0.05 in T2DM patient’s different tissues namely Pancreas, Skeletal muscle and Adipose tissue.

Among the genes implicated in nephropathy complications, 151 genes were differentially expressed in T2DM patients. These include 122 genes in pancreas, 26 genes in adipose tissue and 12 genes in skeletal muscle. The bar plots of the differentially expressed genes were generated to provide a graphical view of normalized gene expression in individual samples in terms of MAD modified Z-scores of diabetic and non-diabetic patients’ (Fig. 6). The y axis in the bar plots represents the normalized expression value whereas ‘x’ axis describes the samples. Additionally, differently expressed genes with P Value < 0.05 were also displayed liberally with an aim to benefit future studies. Genes *ATF4*, *CCL5, EDNRA, SGK3, IGF2, SLC2A1, SOCS3, SRC* and *TIMM44* were differentially expressed in adipose and pancreas. 14 differentially expressed genes *FGF21, IL18, RBP4, ANGPTL2, MIF, VEGFA, ANGPTL4, APOC1, CTGF, CXCL10, GAS6, GREM1, IFNG, IGF2, NGF, REG1A* and *TIMP2* ^136–138, 140, 141, 143–158^ are annotated as secreted and their product proteins are reportedly present in plasma. Four of these genes *VEGFA, TIMP2* and *ANGPTL2* had FC values either >2 or <0.5.

#### Neuropathy

Among the genes implicated in neuropathy complications, 39 genes were differentially expressed in T2DM patients’. These include 32 genes in pancreas, 7 genes in adipose tissue and 3 genes in skeletal muscle. *IGF2* and *TSPO* were differentially expressed in adipose and pancreas. *IRAK1* was differentially expressed in pancreas and skeletal muscle. Nine differentially expressed genes *LEP, MIF, VEGFA, CXCL10, IFNG, IGF2*, and *NGF* are annotated as secreted and their product proteins are also reportedly present in plasma^139, 143, 144, 148, 150, 153–156^. One of these genes, *VEGFA* had FC values either >2 or <0.5.

#### Retinopathy

Among the genes implicated in retinopathy complication 46 genes were differentially expressed in T2DM patients, 36 genes in pancreas, 9 genes in adipose tissue and 4 genes in skeletal muscle. Genes *ATF4, IFG2* and *SOCS3* were differentially expressed in adipose and pancreas. There are 10 differentially expressed genes *FGF21, IL18, INS, RBP4, TIMP1, VEGFA, ANGPTL4, CTGF* and *IGF2* annotated as secreted and their product proteins are also reportedly present in plasma^136–140, 145, 146, 149, 155^. Two of these genes *VEGFA* and *TIMP1* had FC values either >2 or <0.5.

#### Risk factors

We investigated the interaction of risk factors, namely, obesity, inflammation, diet and stress, with the genes of T2DM associated complications, which are differentially expressed in T2DM patients’ 3 tissues. The results are shown in Supplementary Figure 1.

In Pancreas, obesity topped the list with the number of differentially expressed genes (DEG) associated with the complications nephropathy and neuropathy, cardiovascular, and atherosclerosis whereas in case of retinopathy, inflammation topped the list (Supplementary Figure 1a).

In Adipose tissue, obesity topped the list with the number of DEG associated with nephropathy, atherosclerosis, whereas in neuropathy and retinopathy, stress and inflammation are at the top and in cardiovascular complication, diet and inflammation topped the list (Supplementary Figure 1b).

In Skeletal muscle, the number of DEG is less compared with Adipose and Pancreas. It is noteworthy that obesity topped the list with the number of DEG with nephropathy (Supplementary Figure 1c).

### Mixed evidence on genes with passage of time

6 genes *ACE*, *APOE*, *VEGFA*, *TNFRSF11B*, *FABP2* and *VWF* had mixed evidences and observations. In general, the associations of these genes were investigated multiple times. The results are presented in Supplementary Table 4b. It is evident that with time the associative evidences change however in some cases they also get reinforced. For example in the case of *ACE*’s role in cardiovascular, there is positive evidence in 1995 and 2003 but in 2005 in Chinese population a negative evidence was observed. On the other hand the role in Nephropathy, in 1995 there was negative evidence overturned in 2005 and later supported in as recent as 2015. Another noteworthy example is in case of *VEGFA*. The role of *VEGFA* in Retinopathy and Nephropathy was negative in several populations but reported positive in Caucasian (Retinopathy) in the years as recently as 2013–2015. Likewise in the case of *TNFRS11B*, negative evidences were documented in the years 2011 and 2013 but overturned in 2015 in its association with Atherosclerosis. These mixed observations appear most likely due to population specific effects and therefore our genes collection using a greedy approach offers resource for further investigation in a population specific manner. It is also noteworthy that the genes *ACE, APOE, FABP2, TNFRS11B, VEGFA* and *VWF* are targets of many commonly used drugs. The *CLOCK* gene was differentially expressed in both adipose tissue and pancreas in different studies.

### Transcription factor binding sites and miRNA binding sites

We sought to decipher the RNA regulatory entities including transcription factor binding sites and miRNA target sites using RegRNA 2.0. Of 650 genes, 340 genes had 1351 sequence variants. Among the remaining genes, 306 genes had no variants. In case of 4 genes GGT2*, NYS3, SAA@* and *SERPI3* RefSeq DNA sequences were not available.

There were 1039 miRNA target sites encompassing 1272 variant sequences of 340 genes and 298 genes without variant sequences. There were 654 transcriptional regulatory motifs encompassing 1337 out of 1351 variant sequences of 340 genes and 268 genes without variant sequences. Of 7 genes (*AGER, TNFRSF11B, CRK, PON1, ADIPOQ, CRP* and *NOS3*) common to all complications, 3 genes *(HMGB1, PON1* and *TNFRSF11B)* have no variant sequences and 4 genes *(NOS3, CRK, AGER* and *ADIPOQ)* have 17 variant sequences. 170 Transcriptional regulatory motifs and 6 miRNA target sites are present in all the 3 genes *(HMGB1, PON1* and *TNFRSF11B)* without variant sequences. In the case of remaining 4 genes with 17 variant sequences there were 262 Transcriptional regulatory motifs and 43 miRNA target sites.

Among 9 genes *(TNF, IGF2, TGFB1, IL1B, IL6, INS, GHR, APOE* and *EGF)* with wide functional roles, 5 genes *(IL6, INS, TGFB1, IL1B* and *TNF)* had no variant sequences and 4 genes *(APOE, EGF, GHR*, and *IGF2)* had 25 variant sequences. The genes without variant sequences had 181 Transcriptional regulatory motifs and 19 miRNA target sites. Among 25 variant sequences of 4 genes there were 312 Transcriptional regulatory motifs and 38 miRNA target sites. In general, genes with variant had larger numbers of transcriptional regulatory motifs and miRNA target sites indicating larger regulatory space for these genes.

## Discussion

T2DM, the most common form of diabetes principally arising due to insulin resistance and reduced insulin activity is a risk for several complications, namely atherosclerosis, neuropathy, nephropathy, retinopathy and cardiovascular diseases. The principal driver is the exposure of the cells, tissues and other proteins to hyperglycaemic conditions. Our goal in this work was to organize the dispersed information on the genes reportedly associated with complications in T2DM conditions accumulating in the literature. The T2DiACoD houses the compiled information and is made available to users to facilitate ongoing research in this area. Compared with other repositories developed so far towards similar ends, T2DiACoD is more comprehensive encompassing highest number of genes up to date, including information on T2DM complications and is devoid of false positive evidences because of inclusion of a final manual curation step. We believe a combined approach including automated text mining followed by final manual curation would serve better for data mining from large corpii.

The information from the literature have been collected from various sources, namely candidate gene studies, GWAS, miRNAs associated with T2DM and differential expression analysis of genes in T2DM patients’ tissues - pancreas, skeletal muscle, liver and adipose from publicly available data repositories.

The identification of genes studied either in a single or in multiple years bring out the significance of the gene in the particular disease. It is evident that in all complications the genes studied in multiple years outnumber those studied in a single year by 1.06–4.16 folds except in neuropathy, where the numbers in both categories are nearly equal. This analysis aids in recognition of the importance of the gene, namely, whether the gene is being studied for further information or no further analysis are required to illuminate the implication of this gene in the complication. Among the 23 drug targets, 13 have been studied in multiple years, two have been studied in a single year. In case of markers or biomarkers, 2 genes each were studied in multiple years as well as in a single year.

If a gene had high BWI several years ago but later not reported, then its significance may have been lost due to multiple reasons. Given that different genes emerge as significant in different populations such patterns are likely. Genes reported in multiple years usually are considered as reference mentioned in background or in comparison in results. It is worth noting the context of the genes reported in multiple years to guide future studies.

The GO analysis elaborates the distinctness of each complication associated with T2DM. Further it is apparent from the GO enrichment map that atherosclerosis and cardiovascular maps are similar and so are the maps of retinopathy and neuropathy. The nephropathy enrichment map appears distinct with chemokine activities. These results could inform future studies in regard to developing new therapeutic approaches by targeting the prominent pathways.

The genes *AGER, TNFRSF11B, CRK, PON1, ADIPOQ, CRP* and *NOS3* are associated with all 5 major complications of T2DM but none of them attained fold change (FC) either >2 or <0.5 in any of the T2DM patients. It is probable that these genes perhaps play mediatory role as opposed to providing driving motive force. The added feature of T2DiACoD is to enlist the differentially expressed genes involved in the pathogenesis of complications in T2DM patients’ tissues thereby reporting up-regulated and down-regulated genes. These observations illuminate the progress of complications in T2DM patients’ tissues and could inform future studies designed to monitor such progression.

A particularly important data gained from this exercise is the involvement of multiple tissues with respect to differential gene expression of a few genes associated with T2DM complications. It is probable that the differential regulation in multiple tissues may underlie the debilitating complications of T2DM. When considered at high FC (either >2 or <0.5), the differentially expressed genes associated with T2DM complications was most observed in skeletal muscle. However, when all differentially expressed genes are considered that were statistically significant, pancreas tissue topped the list followed by adipose and skeletal muscle. These observations show that while the focus of T2DM complications are at the skeletal muscle tissues, a great number of changes in gene expression take place in pancreas and adipose tissues. The liver appears particularly aloof from these noticeable changes. Although the methods of observing gene expression differences are largely statistics based, it is evident from this work that multiple tissues are involved at apparently variable extents.

It is noteworthy that about a quarter of the protective genes (19 out of 63) are differentially expressed in T2DM patients’ tissues. These observations indicate that the influence on the differential expression of protective genes already starts in T2DM conditions. It is noteworthy that 29 of the genes associated with T2DM in various populations were observed as differentially expressed in T2DM patients’ tissues. These observations while highlighting the immense beneficial contribution of GWAS are also informative in that the gene expression differences between T2DM patients and healthy controls likely depend on additional as yet unknown factors that are likely variable. Among the differentially expressed genes *VEGFA* evidently appears striking and is associated with several complications of T2DM. This information emerging from the integrative approach used in this work could benefit further therapeutic approaches.

Another principal goal in our work was to mine the information of multiple tissues involvement and presence of the associated gene products in plasma. It is noteworthy that a minority of associated genes were differentially expressed in multiple tissues and a minority of genes were reported as detected in plasma. These observations and value added information could serve for marker development in future well designed studies. The differential expressed genes and their product proteins of *TIMP1, ANGPTL2, VEGFA*, and *TIMP2*, reportedly present in plasma could be measured in different stages of T2DM complications and possibly offer information for clinical interventions.

The RegRNA 2.0 data analysis predicted that nearly half of the T2DM genes associated with complications had a large number of variants with proportionately large number of transcription factor binding sites and miRNA binding sites. Although only 34 T2DM associated miRNA targets overlapped with the genes in T2DiACoD database, it is quite probable that a large number of miRNAs may be involved in gene regulation system. This appears to increase the complexity of the entire system of genes underlying the T2DM complications. These data serve to highlight the complex nature of these disorders. However, it is noteworthy that a handful of miRNAs and genes have been identified for therapeutic effects.

Among the risk factors considered in this work, are obesity, inflammation, stress and diet and their interactions with the genes of T2DM associated complications. The major share of differentially expressed genes in T2DM patients as observed here is contributed by pancreas tissue followed by adipose and skeletal muscle tissues. Obesity is clearly a dominant risk factor interacting with the genes of T2DM complications followed by inflammation, diet and stress to variable extents. These results indicate the likelihood of developing a complication given the tissue of measurement of differentially expressed genes and the contributing risk factor. However, it is to be noted that the interaction of the risk factor with a given gene and the association of the same gene with T2DM complication were usually observed from different reports. Thus, these indications provide leads for designing specific future studies.

Thus it is apparent that obesity and inflammation play major role in the development of T2DM and its associated complications and therefore therapeutic regimens may be targeted towards ameliorating these effects. The control of obesity is recommended in the MedlinePlus^159^ site also.

## Conclusions

Several points have emerged from our integrative analysis. (1) We believe a combined approach including automated text mining followed by final manual curation would serve better for data mining from large corpii. (2) It is evident that in all complications the genes studied in multiple years outnumber those studied in a single year by 1.06–4.16 folds except in neuropathy, where the numbers in both categories are nearly equal. This trends analysis shows that many genes are studied by different groups in multiple years meaning that the genes are reliably treated. (3) Among the 23 drug targets, 13 have been studied in multiple years, two have been studied in a single year. In case of markers or biomarkers 2 genes each were studied in multiple years as well as in a single year. Thus majority of the drug targets receive importance in investigations. (4) Genes reported in multiple years usually are considered as reference mentioned in background or in comparison in results. It is worth noting the context of the genes reported in multiple years to guide future studies. (5) It is apparent from the GO enrichment map that atherosclerosis and cardiovascular maps are similar and so are the maps of retinopathy and neuropathy. The nephropathy enrichment map appears distinct with chemokine activities. (6) A few genes *ACE2*, *ADCYAP1*, *HDAC4*, *NCF1*, *NFE2L2*, *OSM*, *SMAD1*, *TGFB1*, *BDNF*, *SYVN1*, *TXNIP*, *CD36*, *CYP2J2*, *NLRP3*, where the details of protective role are known have been described are displayed on the website. (7) Among the differentially expressed genes, *VEGFA* evidently appears striking and is associated with several complications of T2DM. This information emerging from the integrative approach used in this work could benefit further therapeutic approaches. (8) The identification of differentially expressed genes associated with T2DM and its complications in T2DM patients compared with normals indicate that the conditions for setting in of these complications arise in T2DM state already. (9) These observations indicate that the influence on the differential expression of protective genes already starts in T2DM conditions. (10) Obesity is clearly a dominant risk factor interacting with the genes of T2DM complications followed by inflammation, diet and stress to variable extents.

## Material and Methods

All text analytics were carried out in R 3.0.1 and the CRAN package pubmed.mineR^160^. Additional packages^161^ were also used to supplement. An R package wordcloud was used for viewing terms in their order of occurrence frequencies.

### Text mining and curation

#### Classification, summarization and additional data extraction

A total of **4,46,438** abstracts were extracted using the keyword “diabetes” from the PubMed database till 30^th^ November 2016. This corpus is referred to as primary corpus. Wordcloud analysis using the R package “wordcloud“^162^ of the top ranking words extracted using the R package pubmed.mineR revealed that among the complications in diabetic conditions, the top ranking terms were atherosclerosis, neuropathy, nephropathy, retinopathy and cardiovascular and therefore we sub-classified the primary corpus into these 5 sub-corpora using the package pubmed.mineR^160^.

#### Population Study

Among the ethnic population terms, the frequently occurring terms were Indian (excluding Indian Americans), Japanese, Chinese, Americans (African Americans, Indian Americans, White Americans and Asian Americans), European, and Mexican. We used these terms for sub-classification of the primary corpus. The complete information about the gene including SNP information in different populations was obtained using the package NCBI2R^163^. In addition, we collected SNP IDs (rsIDs), their genomic position, odds ratio (OR), and reported p-values in GWAS.

#### Entity recognition and their relationships

Towards a comprehensive approach for gene data mining from literature texts we used sentence tokenization function of pubmed.mineR to extract sentences with co-occurrence of the entities, namely, gene symbols including official symbols recommended by the Human Gene Nomenclature Committee’s (HGNC), other symbols (aliases, previous symbols), alternative names from UniProt and gene name from HGNC and the disease complication terms and their aliases, using sentence tokenization of pubmed.mineR. Because the search algorithm uses regular expression based matching, false positives arise due to use of identical acronyms by authors signifying other meanings. Therefore the extracted sentences were examined for ‘proof of association’ or ‘of evidence’ by examining the entities and the relationship between them. The exercise was repeated with risk factors, namely, obesity, stress (oxidative stress and endoplasmic reticulum stress), diet and inflammation in order to identify lateral associations. These risk factors were top ranking based on their occurrence frequencies. In cases where the assertion was not clear, the entire abstract was examined along with full text wherever available from PubMed central. We used a liberal approach in collecting both ‘firm’ assertions and ‘likely’ indications. [Supplementary: source code].

#### Trends analysis

The Buzz Word Index (BWI) proposed by Jensen *et al*.^164^ provides a convenient approach to obtain the trends in the literature. We computed the BWI value of genes using pubmed.mineR. Buzz words are terms mentioned frequently in a given year compared to that in previous years. In general, if an abstract in a given year contains repeated mention of a gene due to its detailed characterization carried out in the study and reported compared to all the abstracts texts in previous years, the BWI registers a high value. We used a minimum of BWI = 1 in order to collect all the genes attributed with some importance. A single occurrence of a given gene in a given year would have its corresponding BWI register “zero” in that year.

#### Data Collection from other databases

We compared our T2DiACoD with other available databases for T2D and associated complications, namely, DisGeNET and T2D-Db (Table 2) and collected the genes missed in our data mining approach. We obtained two genes for diabetic nephropathy from T2D-Db whereas from DisGeNET we got 16 genes for diabetic nephropathy and 10 genes for diabetic retinopathy. Other genes in these databases were false positives verified by examining the evidences and references they reported and therefore they were excluded.

**Table 2:** Comparative statistics of available resources for T2DM complications.

Database	Description	Complications	Genes	Year	References
T2DiACoD	A gene database of Type 2 Diabetes Associated Complications	Nephropathy Neuropathy Retinopathy Cardiovascular diseases	650	2016	This Work
DisGeNET	Database of gene-disease association	Nephropathies Neuropathies Retinopathies Cardiomyopathy diseases	705^*^	2010 (updated in September 2017)	36
T2D-Db	Reports molecular factors involved in pathogenesis of T2D	Nephropathy Neuropathy Retinopathy Cardiovascular diseases	83	2008 (last updated in Jan-2009)	37
T2D knowledge portal	Database of DNA sequence, functional and epigenomic information, and clinical data from studies on type 2 diabetes and complications	—	—	2015 (last updated in May-2017)	38

*Genes are either associated in Type 1 Diabetes or are false matches (included genes without strong evidences and with negative evidence i.e. co-occurrence of disease and gene in a statement). DisGeNET has collected data from different sources i.e. UniProt, ClinVar, Orphanet, The GWAS catalog, CTD, RGD, MGD and literature data from GAD, LHGDN and BeFree.

#### Web server development

The web interface of T2DiACoD was developed using PHP 5.6.3 and HTML. The consolidated data were entered in MySQL 5.0.11 tables using XAMPP (v3.1.0). XAMPP is an open source cross platform package. It includes MariaDB (database), Apache 2.4.10 (server application) and PHP (scripting language). Data sets can be downloaded as Excel sheets.

### Database structure and content

T2DiACoD database structure consists of diabetic complications, ethnicity (population) studies including GWAS in T2DM, and Drug targets of recent drug development from the literature. Differentially Expressed genes in patients’ and normals’ tissues including adipose, pancreas, and skeletal muscle and miRNAs with target in T2DM and its complications are included. The risk factors included are obesity, inflammation, diet and stress (oxidative stress & endoplasmic reticulum stress). In the case of complications in T2DM cases, we considered atherosclerosis, cardiovascular disease, nephropathy, neuropathy and retinopathy. The information on genes obtained from the respective sub-classified corpora were cast into three tables: (i) Gene information: gene symbol, gene name, synonyms, chromosome number, homologene id in a given complication (ii) NCBI Gene database, (iii) Summary of individual genes: Locus id, gene symbol, gene name, synonyms, Chromosome, Homologene id and observation reporting evidence, and the corresponding PubMed ID (PMID) of the report. The population block contains genes associated with diabetes in various populations and sub-classified as reported and includes SNP ids (rsIds), risk allele, chromosome number, chromosome position, functional class, p-value, odds-ratio and PMID.

#### Expression tab

It consists of graphical view (histogram display) of the differential gene expression in adipose, pancreatic, and skeletal muscle tissues in human patients’. The raw (.CEL file format for affymetrix data or BeadStudio output file format for illumina data) microarray gene expression data for different tissues were accessed from the NCBI GEO^165^. We used different combinations of keywords such as ‘type 2 diabetes AND pancreas’, ’type 2 diabetes AND liver’, ‘type 2 diabetes AND skeletal muscle’, ‘type 2 diabetes AND adipose’ for data retrieval. We collected a total of 11 datasets (GSE29226, GSE40234, GSE16415, GSE23343, GSE15653, GSE25724, GSE38642, GSE20966, GSE29221, GSE25462, GSE12643) for all the available tissues studied in different populations. The affymetrix data were pre-processed using the *affy* package^166^. The .CEL files for each GSE id was read using ReadAffy() and subsequently we performed the background correction and computed the expression using the robust mean average (rma()) function with ‘normalize = FALSE’, which computes the expression and performs the background correction. The Illumina data were pre-processed using *lumi* package^167^ of Bioconductor. The lumi package was used to read the illumina BeadStudio output file for each GSE id using the lumiR() function followed by background correction and computed the expression using the robust mean average (rma()) function with ‘normalize = FALSE’, which computes the expression and performs the background correction. Subsequently, the expression of probes with detection p-values < 0.05 was retained to eliminate low signal intensity genes. The pre-processed data was transformed individually into standard normal using the modified Z-score method based on median absolute deviation (MAD)^168^. MAD measures the variability in the sample about its median. Each dataset was pre-processed and normalised individually (Supplementary Table 5).

$${{\rm{MAD}}}_{{\rm{j}}}=\text{median}(|{{\rm{X}}}_{{\rm{i}}}\,\mbox{--}\,{\rm{median}}({{\rm{X}}}_{{\rm{j}}})|)$$

$${M}_{i}=\frac{0.6745{X}_{i}-median\,({X}_{j})}{MA{D}_{j}}$$

where, X_i_ = data element of the i^th^ row

X_j_ = median of the j^th^ column

MAD_j_ = Median Absolute deviation of j^th^ column

M_i_ = Modified Z-score

Expression of duplicated genes were averaged using the CollapseRows() function of whole genome co-expression network analysis (WGCNA) R package^169^. The rows containing excessive amount of missing data (90% or more) were omitted. The differentially expressed genes of the normalized data were computed between non-diabetic and diabetic human patients’ tissues (Supplementary Figure 2[a-i]). The limma package^161^ was used to compute differential expression. Empirical Bayes moderated t-statistics test was applied to test each individual contrast equal to zero. The **topTable()** function was used to rank genes in order of evidence for differential expression. The genes with P-adjusted (Benjamini and Hochberg) values < 0.05 were selected as differentially expressed genes and their P Values were also noted.

#### miRNA block

miRNAs are small non-coding RNA molecules of 20–25 nt that negatively regulate translation of the target mRNAs by binding to the 3′UTR region^170^. Several miRNAs have been identified that are likely involved in T2DM and their complications^170^. The miRNAs associated with T2DM were identified from the literature along with their corresponding target genes. Subsequently, the targets of these miRNAs were mapped with the gene list of T2DM complications to identify miRNAs with potential for regulation of genes associated with T2DM complications.

#### Functional enrichment analysis

To infer potential biological significance of the reported genes we have used **DAVID** (Database for Annotation, Visualization and Integrated Discovery)^171^. It provides functional annotation and analysis of gene list. The lists of genes were submitted to functional annotation in DAVID to analyze the Gene Ontology (GO), protein domains and pathways. The relevant GO terms associated with input gene list were extracted and analyzed. The GO structure mainly contains three categories, namely, Biological Process (BP) for biological goals, Molecular Function (MF) describing functional roles and Cellular Component (CC) indicates the cellular location where gene products are active. The output table was obtained with the following information: Annotation Cluster, Enrichment Score, Category, Term, Count, % (involved genes/total genes), PValue, Genes, List Total, Pop Hits, Pop Total, Fold Enrichment, Bonferroni, Benjamini and FDR. P-Value (calculated by Fishers’ exact test) as well as FDR< = 0.05 were considered strongly enriched in the annotation categories. The cytoscape plugin EnrichmentMap was used for graphical display of GO terms^172^. EnrichmentMap is a visualisation method for the gene set enrichment results. In the graph, nodes represent gene set involved in the GO term and edges represent the extent of overlap of genes between the two gene sets connected by them. Node size and edge width are proportional to the number of genes. The connection between two nodes describes set of genes that two nodes have in common.

#### Enriched or dilution of positive genes

We selected a sample of 96 genes (Supplementary Table 4a), which were described to be strongly associated with any of the 5 given complications in T2DM through manual examination. We examined whether published reports emerged refuting their associative assertions. Official HGNC symbols as well as alias, previous symbol and full names were considered. In the next steps, the sentences with the word “not” were extracted. The pattern match in the regular expression function was used with the pattern “not” prefixed and suffixed with a space to ensure high specificity. A total of 6 genes with sentences having the word “not” in their observation were identified along with their PMIDs.

As an example: *The Ala54Thr polymorphism of the FABP2 gene is not associated with CHD, markers of the metabolic syndrome, or the fatty acid profile of serum lipids in Finnish CHD patients.PMID:12189904*. For the same gene the positive evidence in our database is *FABP2 confers susceptibility to renal disease in type 2 diabetic patients.PMID:16249461*.

#### RegRNA Analysis

Functional RNA motifs can be identified in input mRNA sequences using the RegRNA 2.0 integrated web server^173–180^. It provides information on Splice sites (donor site, acceptor site, Splicing regulatory motifs (ESE, ESS, ISE, ISS elements)^173^, Polyadenylation sites, Transcriptional motifs (rho-independent terminator, TRANSFAC)^174^, Translational motifs (ribosome binding sites), UTR motifs (UTRsite patterns)^175^, mRNA degradation elements (AU-rich elements), RNA editing sites (C-to-U editing sites), Riboswitches (RiboSW)^176^, RNA *cis*-regulatory elements (Rfam, ERPIN)^177^, similar functional RNA sequences (fRNAdb)^178^, RNA-RNA interaction regions (miRNA, ncRNA)^178^, User defined Motif (RNAMotif)^179^, Miscellaneous information (open reading frame, GC-ratio, RNA accessibility)^180^ Sequences greater than 12 kbp size were divided into two equal halves and RegRNA analysis carried out and considered in overall counts.

#### Sub-cellular location

Subcellular locations for all the genes in T2DIACoD were collected from the UniProt^181^.

#### Data access for users

Users can access T2DiACoD by mouse click on the complications in diabetic conditions (atherosclerosis, diabetic neuropathy, diabetic nephropathy, diabetic retinopathy, cardiovascular disease) and Drug targets. The gene centric information for all the genes enlisted for the corresponding ‘selection’ will be displayed. Additional information on gene ontology, literature reference reporting evidence is also provided. The population studies (Chinese, American, Indian, European, Japanese, and Mexican) link enables the user to access the single nucleotide polymorphism (SNP) information on the variations investigated in different ethnic populations with T2DM including link to the published references reporting the data.

The expression search tab on the homepage offers graphical display of the differentially expressed genes in different patients’ tissues – adipose, pancreas, and skeletal muscle.

We have provided the expression tables of each gene for four different tissues (Adipose, Skeletal Muscle, and Pancreas) having different sample Ids with their corresponding-value,adj.P-value and fold change (FC) values as well as brief information of microarray data and its sample. The user can also view the expression graph by clicking the expression link from the gene and SNP information data tables. The bar graph displays the gene expression in diabetic cases compared to the non-diabetic controls in different tissues. A liberal selection was adopted including genes differentially expressed at *Padj* < = 0.05 and *p* value < = 0.05 for visual inspection by users and to guide future studies. The miRNA search tab provides information about the miRNAs with their corresponding target gene associated with T2DM and the link to the reference describing evidential support of miRNAs to the pathogenesis of disease.

Instant search option with gene symbol is available to obtain a brief summary of the genes including Locus id, gene symbol, gene name, synonyms, Chromosome, Homologene id and observation reporting evidence with corresponding literature reference PubMed ID (PMID). An Advanced search is also made available to conduct combinatorial queries to fetch the common genes between T2DM associated complex disorders and risk factors, namely, stress, inflammation, diet and obesity known to predispose individuals to T2DM.

## Electronic supplementary material

Supplementary Data

Supplementary Table

Supplementary Table 4

Supplementary Table 5

Supplementary Table 6
