# Supplementary material for: T2DiACoD: A Gene Atlas of Type 2 Diabetes Mellitus Associated Complex Disorders
Source: Sci Rep. 2017 Jul 31;7:6892. doi: 10.1038/s41598-017-07238-0 (PMC5537262; doi:10.1038/s41598-017-07238-0)
Supplement: Supplementary file 1 — Supplementary Data [file 41598_2017_7238_MOESM1_ESM.doc]

**T2DiACoD: A Gene Atlas of Type 2 Diabetes Mellitus Associated Complex Disorders**

Jyoti Rani^&^, Inna Mittal^&^, Atreyi Pramanik, Namita Singh, Namita Dube, Smriti Sharma, Bhanwar Lal Puniya, Muthukurussi Varieth Raghunandanan, Ahmed Mobeen^#^, Srinivasan Ramachandran*^#^

*G N Ramachandran Knowledge of Centre, Council of Scientific and Industrial Research – Institute of Genomics and Integrative Biology (CSIR-IGIB), Room No.130, Mathura Road, New Delhi 110025, India.*

*^#^Academy of Scientific and Innovative Research, CSIR-IGIB South Campus, New Delhi 110025, India.*

^&^ *Both authors contributed equally in this work.*

Corresponding author:

Srinivasan Ramachandran

Room No. 130

CSIR- Institute of Genomics and Integrative Biology

South Campus, Mathura Road

New Delhi 110 025 India

+91-11-29879130, ramu@igib.in, ramu@igib.res.in, [ramuigib@gmail.com](mailto:ramuigib@gmail.com)

**Supplementary Figure 1a**


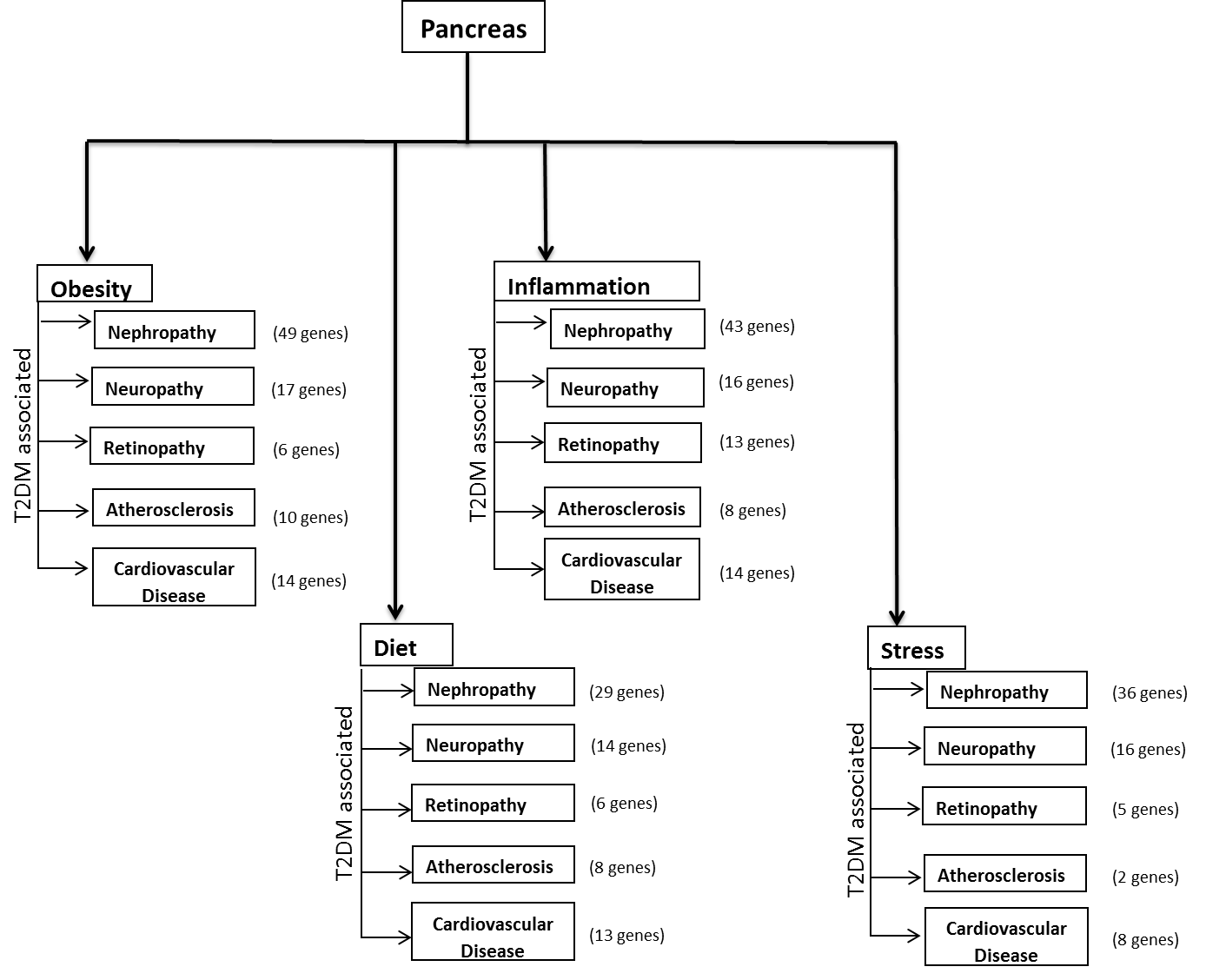


Supplementary Figure 1 : (a) Pancreas. The number of differentially expressed genes (DEG) associated with each of the 5 T2DM complications interacting with the 4 risk factors obesity, inflammation, stress and diet.

# Note that the association of risk factor with a given gene was obtained from the literature mining. This tree representation includes the meta-analysis information.

**Supplementary Figure 1b**


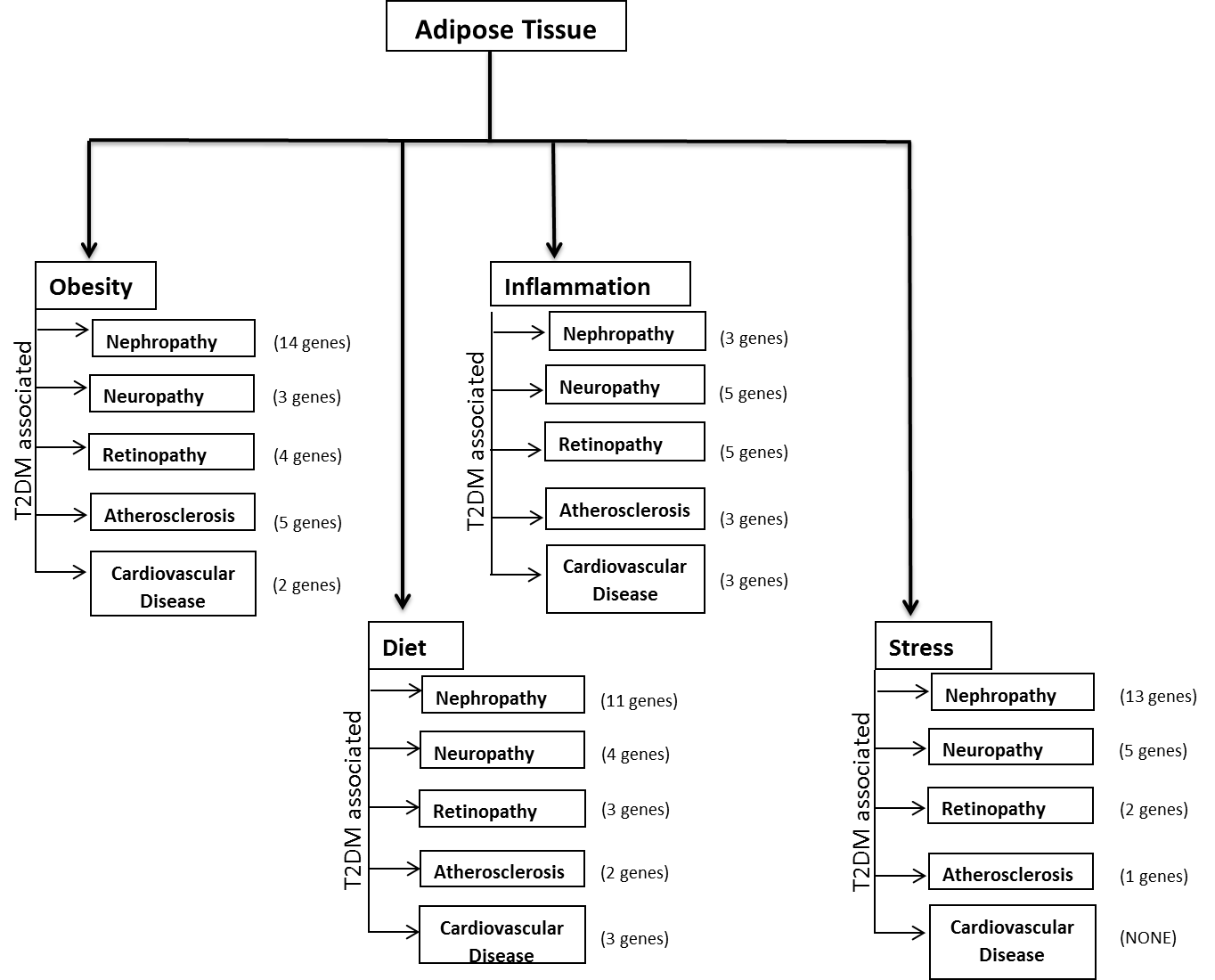


Supplementary Figure 1 : (b) Adipose Tissue. The number of differentially expressed genes (DEG) associated with each of the 5 T2DM complications interacting with the 4 risk factors obesity, inflammation, stress and diet.

**Supplementary Figure 1c**


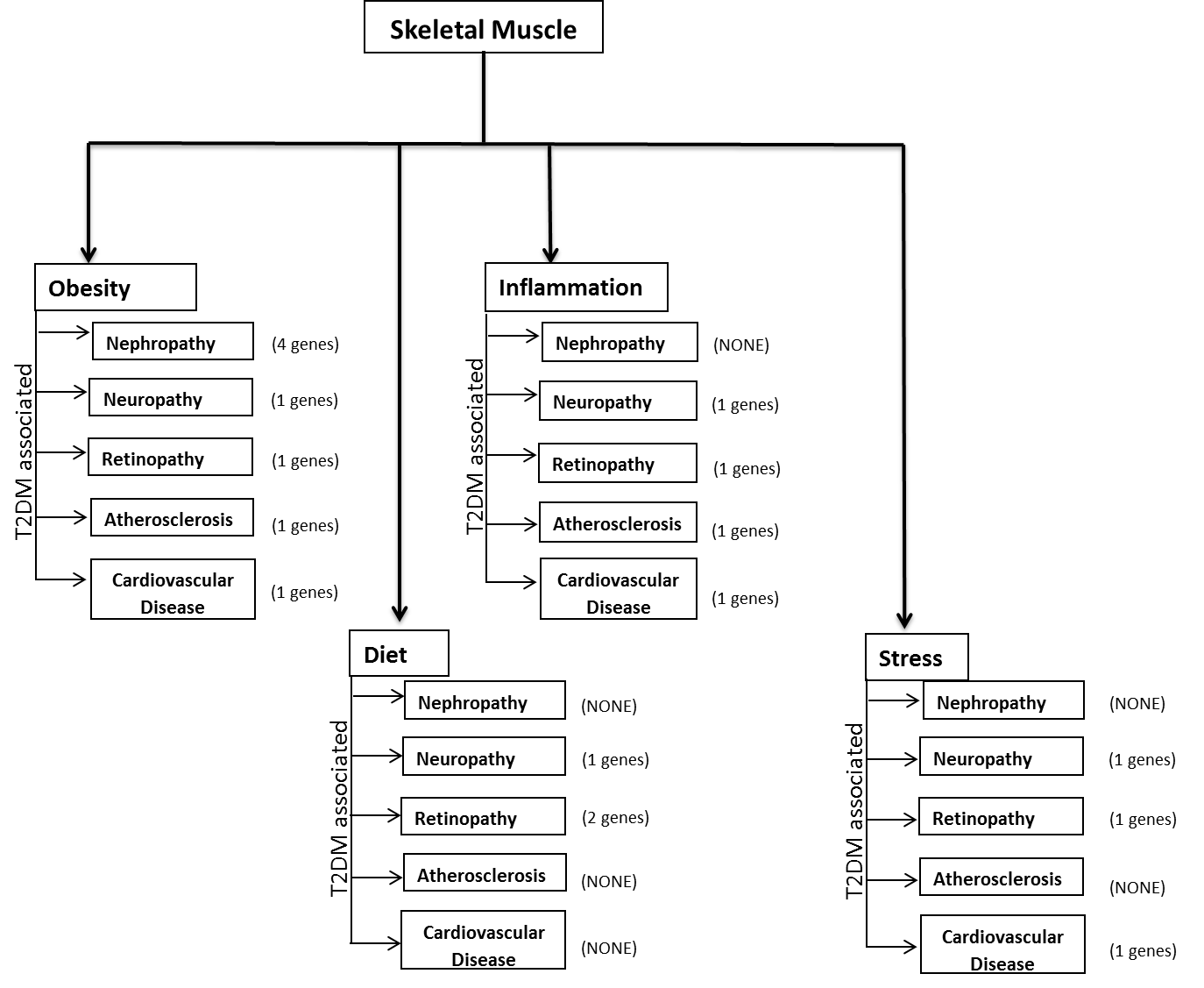


Supplementary Figure 1 : (c) Skeletal Muscle. The number of differentially expressed genes (DEG) associated with each of the 5 T2DM complications interacting with the 4 risk factors obesity, inflammation, stress and diet.

**Supplementary Figure 2a**


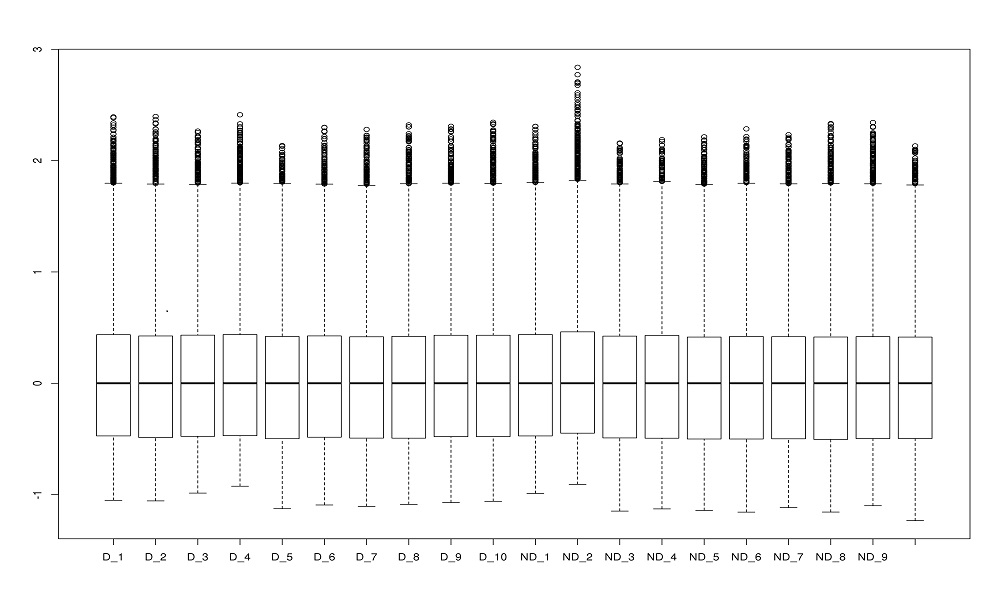


Supplementary Figure 2a: Box plots showing the normalized data of skeletal muscle (GSE12643) samples from T2DM patients (Samples with “D” labels) and controls (Samples with “ND” labels) using median absolute deviation (MAD) method. The horizontal line in the box shows the median of the data.

**Supplementary Figure 2b**


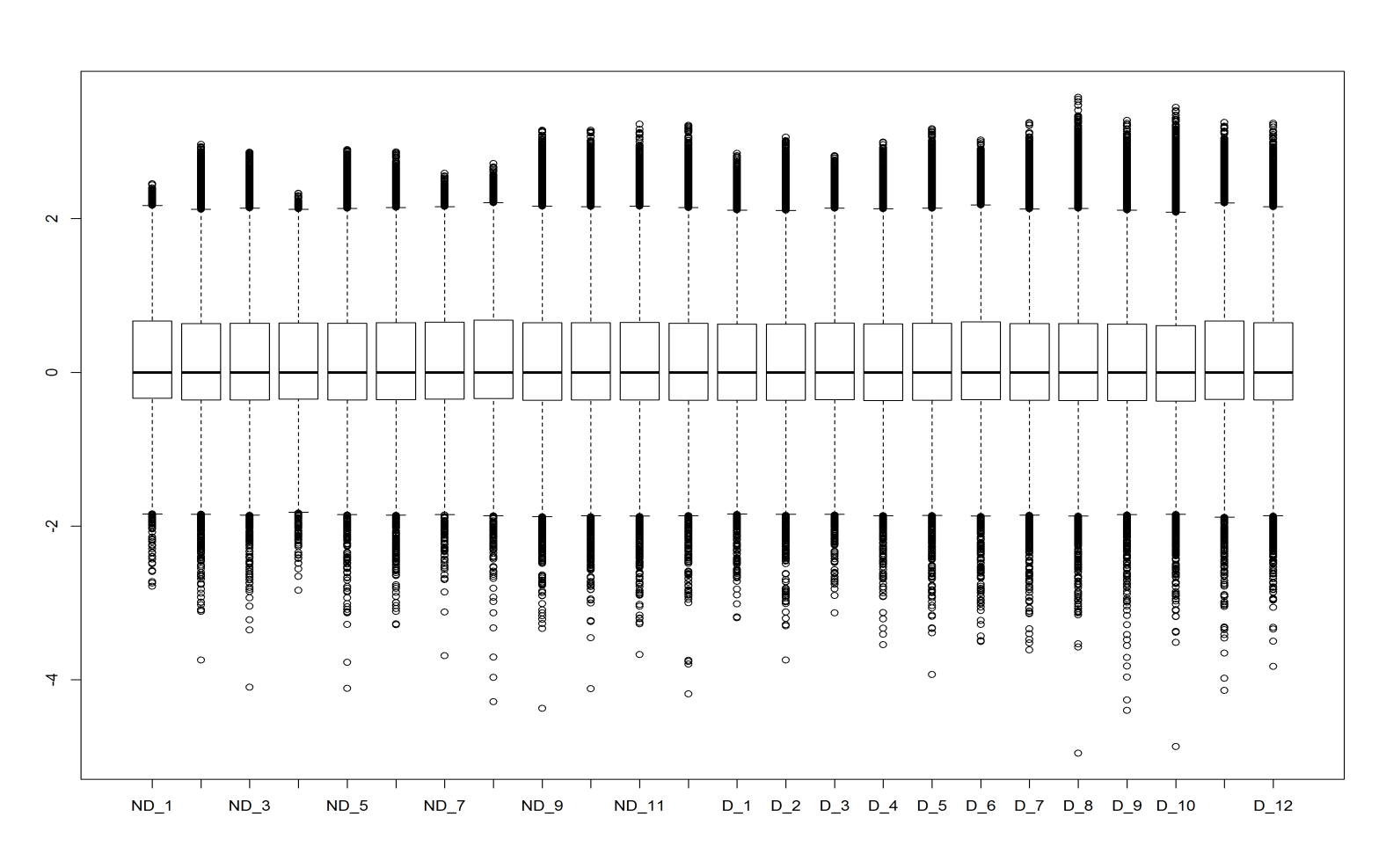


Supplementary Figure 2b: Box plots showing the normalized data of skeletal muscle (GSE29221) samples from T2DM patients (Samples with “D” labels) and controls (Samples with “ND” labels) using median absolute deviation (MAD) method.

**Supplementary Figure 2c**


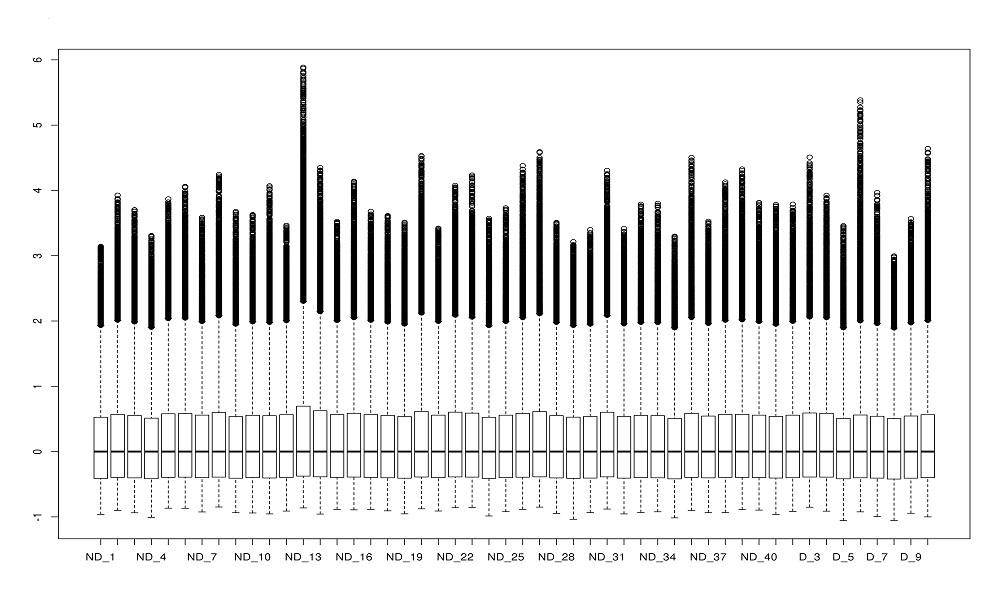


Supplementary Figure 2c: Box plots showing the normalized data of skeletal muscle (GSE25462) samples from T2DM patients (Samples with “D” labels) and controls (Samples with “ND” labels) using median absolute deviation (MAD) method.

**Supplementary Figure 2d**


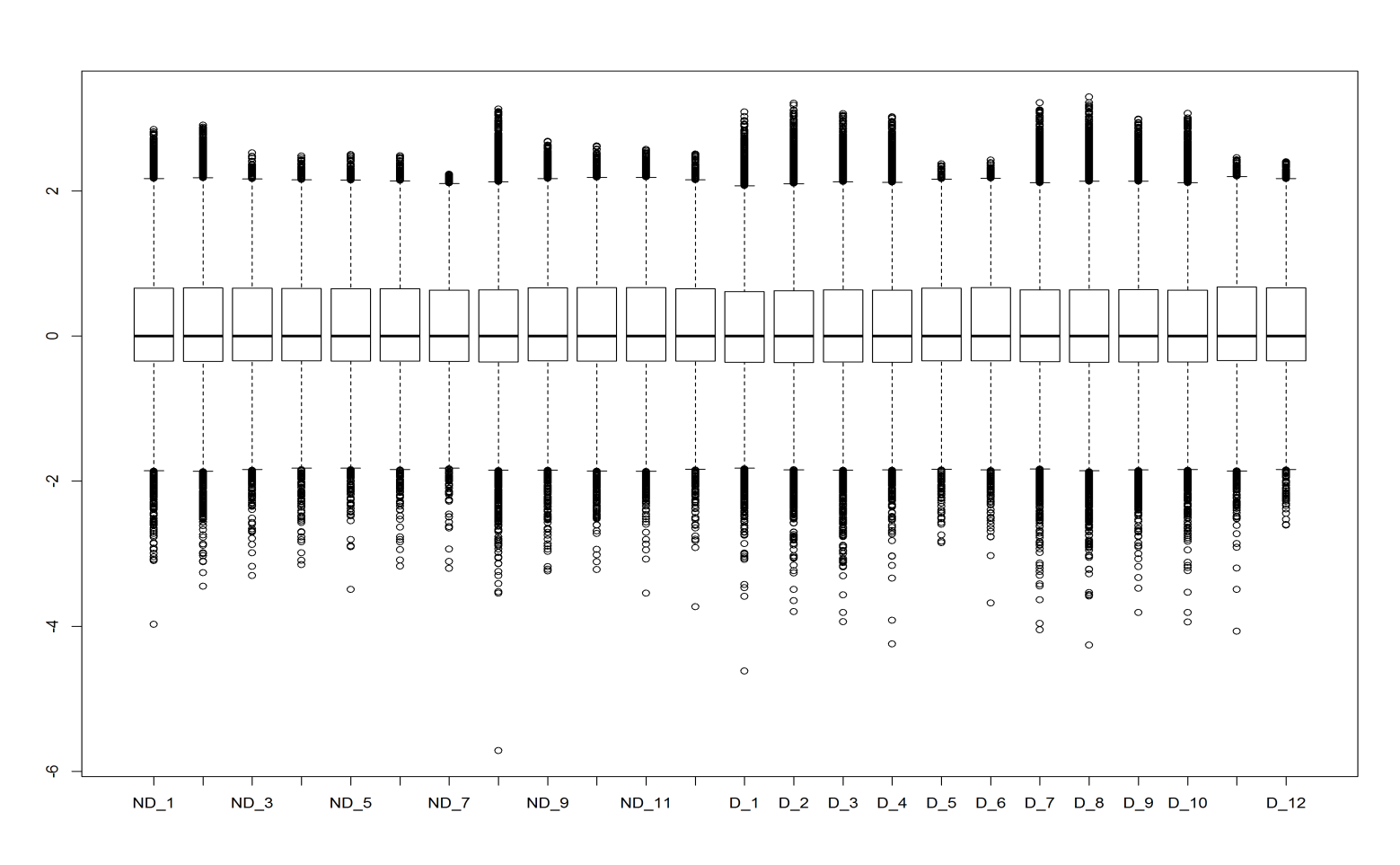


Supplementary Figure 2d: Box plots showing the normalized data of adipose tissue (GSE29226) samples from T2DM patients (Samples with “D” labels) and controls (Samples with “ND” labels) using median absolute deviation (MAD) method.

**Supplementary Figure 2e**


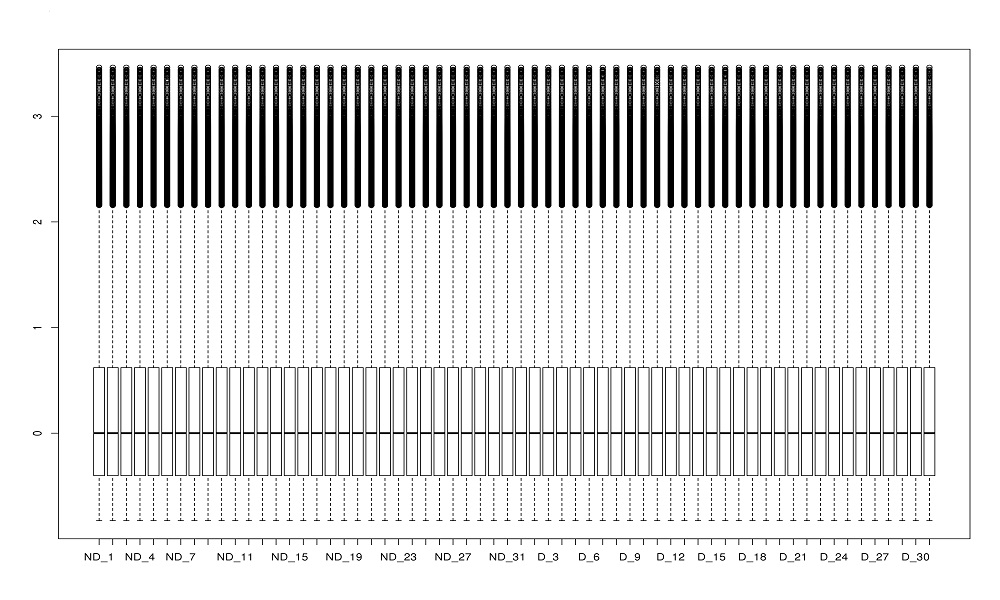


Supplementary Figure 2e: Box plots showing the normalized data of adipose tissue (GSE40234) samples from T2DM patients (Samples with “D” labels) and controls (Samples with “ND” labels) using median absolute deviation (MAD) method.

**Supplementary Figure 2f**


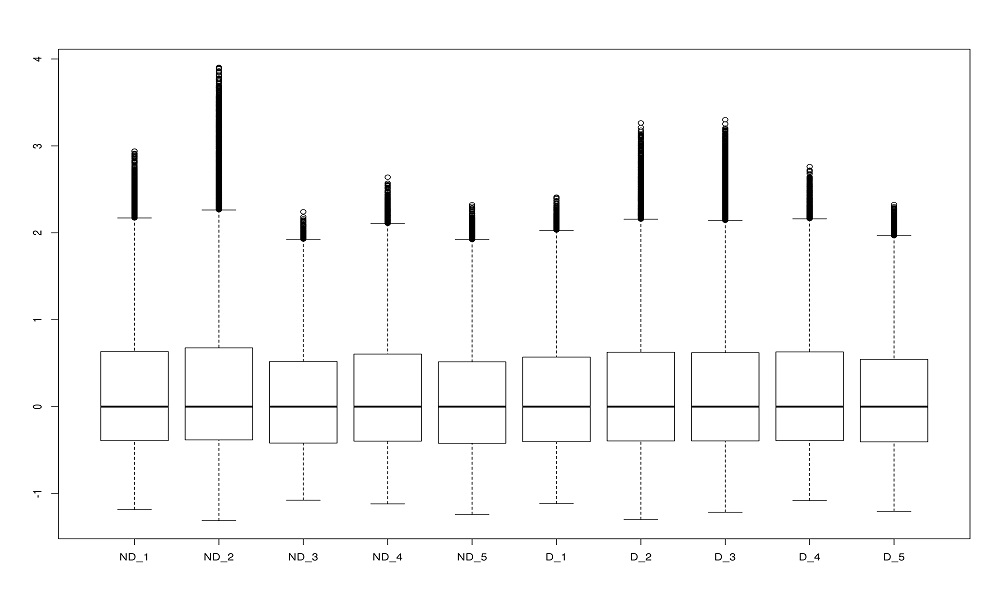


Supplementary Figure 2f: Box plots showing the normalized data of adipose tissue (GSE16415) samples from T2DM patients (Samples with “D” labels) and controls (Samples with “ND” labels) using median absolute deviation (MAD) method.

**Supplementary Figure 2g**


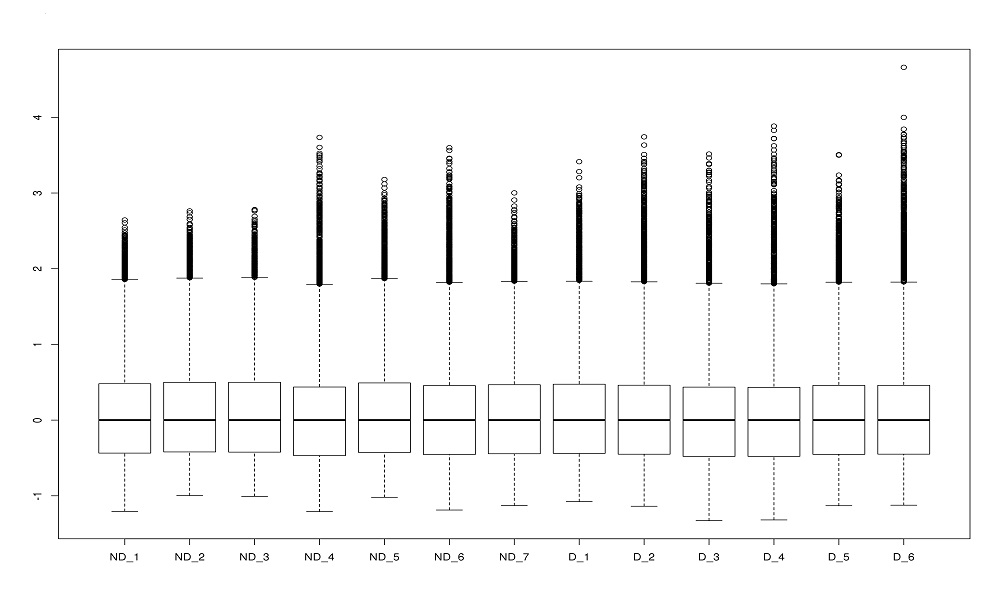


Supplementary Figure 2 g: Box plots showing the normalized data of pancreas (GSE25724) samples from T2DM patients (Samples with “D” labels) and controls (Samples with “ND” labels) using median absolute deviation (MAD) method.

**Supplementary Figure 2h**


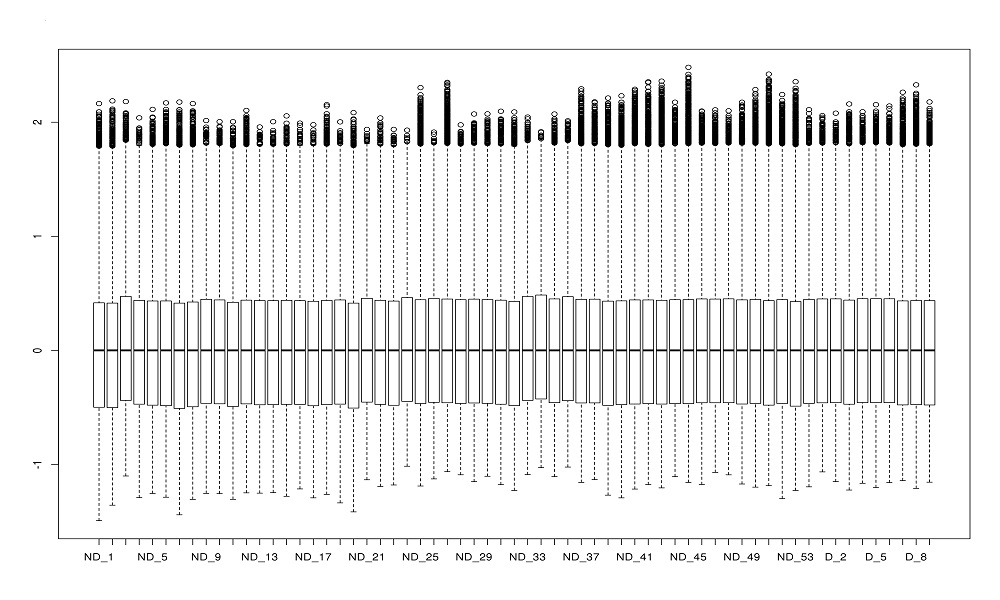


Supplementary Figure 2h: Box plots showing the normalized data of pancreas (GSE38642) samples from T2DM patients (Samples with “D” labels) and controls (Samples with “ND” labels) using median absolute deviation (MAD) method.

**Supplementary Figure 2i**


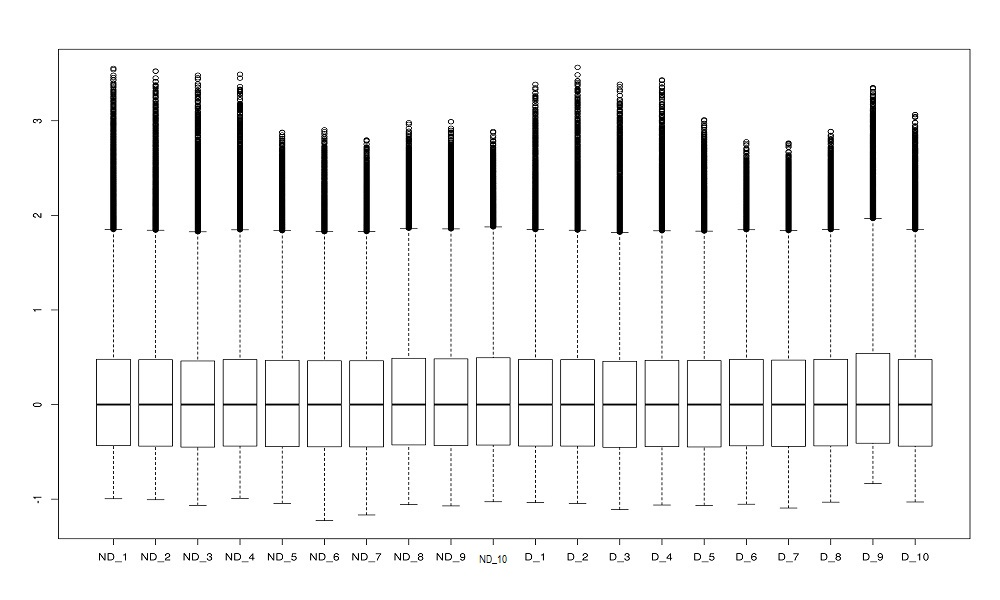


Supplementary Figure 2i: Box plots showing the normalized data of pancreas (GSE20966) samples from T2DM patients (Samples with “D” labels) and controls (Samples with “ND” labels) using median absolute deviation (MAD) method.

**Supplementary Table 2a - Gene Ontology (GO) terms from DAVID found unique for genes implicated in cardiovascular diseases.**

| GO:0042157~lipoprotein metabolic process |
| --- |
| GO:0042632~cholesterol homeostasis |
| GO:0034364~high-density lipoprotein particle |
| GO:0017127~cholesterol transporter activity |
| GO:0043691~reverse cholesterol transport |
| GO:0008203~cholesterol metabolic process |
| GO:0010745~negative regulation of macrophage derived foam cell differentiation |
| GO:0005543~phospholipid binding |
| GO:0033344~cholesterol efflux |
| GO:0033700~phospholipid efflux |
| GO:0055091~phospholipid homeostasis |
| GO:0015485~cholesterol binding |
| GO:0032489~regulation of Cdc42 protein signal transduction |
| GO:0034374~low-density lipoprotein particle remodeling |
| GO:0034375~high-density lipoprotein particle remodeling |
| GO:0005576~extracellular region |
| GO:0005615~extracellular space |
| GO:0046326~positive regulation of glucose import |
| GO:0008201~heparin binding |
| GO:0045944~positive regulation of transcription from RNA polymerase II promoter |
| GO:0019433~triglyceride catabolic process |
| GO:0070328~triglyceride homeostasis |
| GO:0034361~very-low-density lipoprotein particle |
| GO:0042627~chylomicron |
| GO:0008289~lipid binding |
| GO:0001523~retinoid metabolic process |
| GO:0030301~cholesterol transport |
| GO:0006641~triglyceride metabolic process |
| GO:0050750~low-density lipoprotein particle receptor binding |
| GO:0000302~response to reactive oxygen species |
| GO:0016209~antioxidant activity |
| GO:0008134~transcription factor binding |
| GO:0045454~cell redox homeostasis |

**Supplementary Table 2b -** Gene Ontology (GO) terms from DAVID found unique for genes implicated in atherosclerosis.

| GO:0042157~lipoprotein metabolic process |
| --- |
| GO:0042632~cholesterol homeostasis |
| GO:0010745~negative regulation of macrophage derived foam cell differentiation |
| GO:0043691~reverse cholesterol transport |
| GO:0009897~external side of plasma membrane |
| GO:0071222~cellular response to lipopolysaccharide |
| GO:0034364~high-density lipoprotein particle |
| GO:0008203~cholesterol metabolic process |
| GO:0033344~cholesterol efflux |
| GO:0017127~cholesterol transporter activity |
| GO:0010887~negative regulation of cholesterol storage |
| GO:0034380~high-density lipoprotein particle assembly |
| GO:0005543~phospholipid binding |
| GO:0055091~phospholipid homeostasis |
| GO:0005548~phospholipid transporter activity |
| GO:0034375~high-density lipoprotein particle remodeling |
| GO:0005615~extracellular space |
| GO:0005576~extracellular region |
| GO:0042593~glucose homeostasis |
| GO:0034383~low-density lipoprotein particle clearance |
| GO:0032735~positive regulation of interleukin-12 production |
| GO:0070328~triglyceride homeostasis |
| GO:0070508~cholesterol import |
| GO:0019433~triglyceride catabolic process |
| GO:0071356~cellular response to tumor necrosis factor |
| GO:0032760~positive regulation of tumor necrosis factor production |
| GO:0030169~low-density lipoprotein particle binding |

**Supplementary Table 2c -** Gene Ontology (GO) terms from DAVID found unique for genes implicated in diabetic nephropathy.

| GO:0005615~extracellular space |
| --- |
| GO:0005576~extracellular region |
| GO:0006955~immune response |
| GO:0005886~plasma membrane |
| GO:0042157~lipoprotein metabolic process |
| GO:0010745~negative regulation of macrophage derived foam cell differentiation |
| GO:0005887~integral component of plasma membrane |
| GO:0010875~positive regulation of cholesterol efflux |
| GO:0003081~regulation of systemic arterial blood pressure by renin-angiotensin |
| GO:0045944~positive regulation of transcription from RNA polymerase II promoter |
| GO:0006468~protein phosphorylation |
| GO:0022617~extracellular matrix disassembly |
| GO:0032755~positive regulation of interleukin-6 production |
| GO:0045909~positive regulation of vasodilation |
| GO:0006006~glucose metabolic process |
| GO:0008201~heparin binding |
| GO:0032735~positive regulation of interleukin-12 production |
| GO:0060326~cell chemotaxis |
| GO:0007263~nitric oxide mediated signal transduction |
| GO:0031663~lipopolysaccharide-mediated signaling pathway |
| GO:0048015~phosphatidylinositol-mediated signaling |
| GO:0018105~peptidyl-serine phosphorylation |
| GO:0071407~cellular response to organic cyclic compound |
| GO:0014066~regulation of phosphatidylinositol 3-kinase signaling |
| GO:0004672~protein kinase activity |
| GO:0020037~heme binding |
| GO:0008013~beta-catenin binding |
| GO:0000122~negative regulation of transcription from RNA polymerase II promoter |
| GO:0044212~transcription regulatory region DNA binding |
| GO:0006935~chemotaxis |
| GO:0008009~chemokine activity |
| GO:0000165~MAPK cascade |
| GO:0070098~chemokine-mediated signaling pathway |
| GO:0007259~JAK-STAT cascade |
| GO:0090026~positive regulation of monocyte chemotaxis |
| GO:0071347~cellular response to interleukin-1 |
| GO:0032760~positive regulation of tumor necrosis factor production |
| GO:0051770~positive regulation of nitric-oxide synthase biosynthetic process |
| GO:0044130~negative regulation of growth of symbiont in host |
| GO:0001516~prostaglandin biosynthetic process |
| GO:0046934~phosphatidylinositol-4,5-bisphosphate 3-kinase activity |
| GO:0042554~superoxide anion generation |
| GO:0016175~superoxide-generating NADPH oxidase activity |
| GO:0043020~NADPH oxidase complex |
| GO:0006801~superoxide metabolic process |
| GO:0035994~response to muscle stretch |
| GO:0060397~JAK-STAT cascade involved in growth hormone signaling pathway |

**Supplementary Table 2d -** Gene Ontology (GO) terms from DAVID found unique for genes implicated diabetic retinopathy.

| GO:0051092~positive regulation of NF-kappaB transcription factor activity |
| --- |
| GO:0050731~positive regulation of peptidyl-tyrosine phosphorylation |
| GO:0008201~heparin binding |
| GO:0045725~positive regulation of glycogen biosynthetic process |
| GO:0007263~nitric oxide mediated signal transduction |
| GO:0001525~angiogenesis |
| GO:0001523~retinoid metabolic process |
| GO:0031622~positive regulation of fever generation |
| GO:0050661~NADP binding |
| GO:0005179~hormone activity |
| GO:0008083~growth factor activity |
| GO:0050840~extracellular matrix binding |
| GO:0031663~lipopolysaccharide-mediated signaling pathway |
| GO:0046326~positive regulation of glucose import |
| GO:0071682~endocytic vesicle lumen |
| GO:0005125~cytokine activity |
| GO:0020037~heme binding |
| GO:0045776~negative regulation of blood pressure |
| GO:0045909~positive regulation of vasodilation |
| GO:0000302~response to reactive oxygen species |
| GO:0045944~positive regulation of transcription from RNA polymerase II promoter |
| GO:0034617~tetrahydrobiopterin binding |
| GO:0045429~positive regulation of nitric oxide biosynthetic process |
| GO:0005576~extracellular region |
| GO:0005615~extracellular space |

**Supplementary Table 2e - Gene Ontology (GO) terms from DAVID found unique for genes implicated in diabetic neuropathy**

| GO:0005576~extracellular region |
| --- |
| GO:0005615~extracellular space |
| GO:0005886~plasma membrane |
| GO:0005179~hormone activity |
| GO:0050731~positive regulation of peptidyl-tyrosine phosphorylation |
| GO:0005125~cytokine activity |
| GO:0005887~integral component of plasma membrane |
| GO:0000187~activation of MAPK activity |
| GO:0008083~growth factor activity |
| GO:0045429~positive regulation of nitric oxide biosynthetic process |
| GO:0048661~positive regulation of smooth muscle cell proliferation |
| GO:0031093~platelet alpha granule lumen |
| GO:0002576~platelet degranulation |
| GO:0006955~immune response |
| GO:0005184~neuropeptide hormone activity |
| GO:0071222~cellular response to lipopolysaccharide |
| GO:0031663~lipopolysaccharide-mediated signaling pathway |
| GO:0051091~positive regulation of sequence-specific DNA binding transcription factor activity |
| GO:0045348~positive regulation of MHC class II biosynthetic process |
| GO:0043410~positive regulation of MAPK cascade |

**Supplementary Table 3 - The 34 miRNAs implicated in the T2DM and their targets. These targets are also associated with T2DM complications and therefore these 34 miRNAs are likely involved in the T2DM complications.**

| **miRNA** | **Target Gene** | **PMID** |
| --- | --- | --- |
| miR-33a | *ABCA1* | 22315319 |
| miR-103/107 | *CAV1* | 21654750 |
| miR-124, miR29 | *FOXA2* | 22156553, 24722248 |
| miR-192, miR-194 | *HNF1A* | 21294859 |
| miR-802 | *HNF1B* | 23389544 |
| miR-25 | *NOX4* | 21071935 |
| miR-21, miR-152 | *PTEN* | 22956257, 26996529 |
| mir-181a, miR-34a, mir-9, miRNA-9-3p, miR-195 | *SIRT1* | 22476949, 23834033, 21288303, 26459099, 24570140 |
| miR-21 | *SMAD7* | 23292313 |
| miR-144 | *IRS1* | 24497980 |
| miR-155 | *NR1H3* | 23991091 |
| miR-106b, miR-93 | *UCP1* | 23954633 |
| miR-106b, miR-27a, miR-30d | *SLC2A4* | 27165190 |
| miR-17 | *TXNIP* | 26858253 |
| miR-192 | *ALB* | 26881255 |
| miR-194 | *AKT1* | 27163678 |
| miR-199b-5p | *KL* | 26813039 |
| miRNA33b/16 | *INS* | 27301461 |
| miR-51 | *GK* | 27495223 |
| miR-199a-3p | *LEP* | 27279151 |
| miR146 | *IRAK1, TRFA6* | 18633110 |
| miR-328 | *BAAT* | 26900752 |
| miR1276 | *BMP2* | 24418602 |
| miRNA-29 | *FTO* | 26691922 |
| miR-200b, miR-106a | *VEGFA* | 21357793, 24018047 |

**Supplementary - Source Code**

******************************************************************************************************

#Source Code for gene data mining for T2D and its associated five complications from PubMed mining#

library("pubmed.mineR") #to extract genes with observations and PMIDs#

library("openxlsx") #to read xlsx file#

diabetes <- readabs("filename.txt") #Primary corpus of type 2 diabetes. Search keyword in PubMed: type 2 diabetes,T2D,type 2 diabetes mellitus,T2DM

hgnc_gene_table = read.xlsx("hgnc_table.xlsx") # Reading in HGNC gene table downloaded from HGNC site

hgnc_gene_table_1 = hgnc_gene_table[,-1] #Removing the HGNC ids column

#Secondary corpus for five T2D complications

neuro = searchabsL(diabetes,include = c("neuropathy","polyneuropathy"))

nephro = searchabsL(diabetes,include = c("nephropathy","glomerulnephritis","acute kidney disease",”end stage renal disease”))

retino = searchabsL(diabetes,include = c("retinopathy","retinal vascular disease",”DR”))

athero = searchabsL(diabetes,include =c("atherosclerosis","coronary heart disease","atherosclerotic","atherogenic"))

cardio = searchabsL(diabetes,include = c("cardiovascular","ischemic stroke","CHD","coronary heart disease"))

#Observations containing gene symbol (official, alias, previous symbol gene name) and PMID were extracted. All results were manually examined and False positives were removed:

#Diabetic neuropathy. Keywords were based on MeSH (Medical Subject Headings) Unique ID for Diabetic Neuropathy: D003929. neuropathy is a segment of the binomial term diabetic neuropathy and of the trinomial keyword diabetic peripheral neuropathy. mononeuropathy and polyneuropathy were considered as separate keywords. Thus, the search using neuropathy is likely to be sufficient, in principle, to extract the sentences with binomial and trinomial terms. We however used a liberal approach in our keywords terms. Redundant extracts produced during this approach were considered as proof-of obtaining correct data and increase of confidence during manual curation. During manual curation, we focussed on the connection of diabetes with neuropathy (for example, diabetic neuropathy (DN) or diabetic peripheral neuropathy (DPN) or diabetic polyneuropathy). Disambiguation of abbreviations were carried out using the head_abbrev() function of the R package pubmed.mineR. As an example - *In vivo knockdown of ORP150 induced DPN in early  diabetes and exacerbated the DPN after prolonged diabetes, whereas knockdown of CHOP ameliorated DPN in rats with prolonged diabetic.PMID:23988440*

official_fn(hgnc_gene_table_1,neuro,"neuro_",c("neuropathy","diabetic neuropathy","autonomic neuropathy","mononeuropathy","polyneuropathy",”DN”,”DPN”))

alias_fn(hgnc_gene_table_1,hgnc_gene_table,neuro,"neuro_",c("neuropathy","diabetic neuropathy","autonomic neuropathy","mononeuropathy","polyneuropathy",”DN”,”DPN”))

prevsymbol_fn(hgnc_gene_table_1,hgnc_gene_table,neuro,"neuro_",c("neuropathy","diabetic neuropathy","autonomic neuropathy","mononeuropathy","polyneuropathy",”DN”,”DPN”))

names_fn(hgnc_gene_table_1,hgnc_gene_table,neuro,"neuro_",c("neuropathy","diabetic neuropathy","autonomic neuropathy","mononeuropathy","polyneuropathy",”DN”,”DPN”))

#Diabetic Nephropathy. Search keywords based on MeSH Unique ID for Diabetic Nephropathy: D003928. nephropathy is a segment of the binomial term diabetic nephropathy. Thus, the search using nephropathy is likely to be sufficient, in principle, to extract the sentences with binomial terms. We however used a liberal approach in our keywords terms. Redundant extracts produced during this approach were considered as proof-of obtaining correct data and increase of confidence during manual curation. Additional keywords were included based on relevance used in the literature. During manual curation, we focussed on the connection of diabetes with nephropathy (for example, diabetic nephropathy (DN)). Disambiguation of abbreviations were carried out using the head_abbrev() function of the R package pubmed.mineR. As an example -  *Our results indicate that NQO1*2 genotype may increase susceptibility to DN in north Indian subjects with T2DM. PMID:27078674*

official_fn(hgnc_gene_table_1,nephro,"Nephropathy_",c("nephropathy","diabetic nephropathy","glomerulnephritis","acute kidney disease","AKD",”DN”,”end stage renal disease”))

alias_fn(hgnc_gene_table_1,hgnc_gene_table,nephro,"Nephropathy_",c("nephropathy","diabetic nephropathy","glomerulnephritis","acute kidney disease","AKD",”DN”,”end stage renal disease”))

names_fn(hgnc_gene_table_1,hgnc_gene_table,nephro,"Nephropathy_",c("nephropathy", "diabetic nephropathy","glomerulnephritis","acute kidney disease","AKD",”DN”,”end stage renal disease”))

prevsymbol_fn(hgnc_gene_table_1,hgnc_gene_table,nephro,"Nephropathy_",c("nephropathy", "diabetic nephropathy","glomerulnephritis","acute kidney disease","AKD",”DN”,”end stage renal disease”))

#Diabetic Retinopathy. #Search keywords based on MeSH Unique ID for Diabetic Retinopathy: D003930. retinopathy is a segment of the binomial term diabetic retinopathy. Thus, the search using retinopathy is likely to be sufficient, in principle, to extract the sentences with binomial terms. We however used a liberal approach in our keywords terms. Redundant extracts produced during this approach were considered as proof-of obtaining correct data and increase of confidence during manual curation. During manual curation, we focussed on the connection of diabetes with retinopathy (for example, diabetic retinopathy(DR)). Disambiguation of abbreviations were carried out using the head_abbrev() function of the R package pubmed.mineR. As an example -  *In conclusion , our findings suggest that this IGF-I gene polymorphism is associated with an increased risk of diabetic retinopathy. PMID:16873705*

official_fn(hgnc_gene_table_1,retino,"Retinopathy_",c("retinopathy","diabetic retinopathy","retinal vascular disease",”DR”))

alias_fn(hgnc_gene_table_1,hgnc_gene_table,retino,"Retinopathy_",c("retinopathy","diabetic retinopathy","retinal vascular disease",”DR”))

prevsymbol_fn(hgnc_gene_table_1,hgnc_gene_table,retino,"Retinopathy_",c("retinopathy", "diabetic retinopathy","retinal vascular disease",”DR”))

names_fn(hgnc_gene_table_1,hgnc_gene_table,retino,"Retinopathy_",c("retinopathy","diabetic retinopathy","retinal vascular disease",”DR”))

#Atherosclerosis. Search keywords based on MeSH Unique ID for Atherosclerosis: D050197

During manual curation, we focussed on the connection of diabetes with atherosclerosis or other related terms see below in the script. As an example - *The GSTT1-0 genotype and GSTT1-0/GSTM1-0 haplotype might be a potential determinants of susceptibility to  advanced atherosclerosis in patients with type 2 diabetes mellitus. PMID:22330623*

official_fn(hgnc_gene_table_1,athero,"Atherosclerosis_",c("atherosclerosis","atherosclerotic", "atherogenic"))

alias_fn(hgnc_gene_table_1,hgnc_gene_table,athero,"Atherosclerosis_",c("atherosclerosis", "atherosclerotic","atherogenic"))

names_fn(hgnc_gene_table_1,hgnc_gene_table,athero,"Atherosclerosis_",c("atherosclerosis", "atherosclerotic","atherogenic"))

prevsymbol_fn(hgnc_gene_table_1,hgnc_gene_table,athero,"Atherosclerosis_",c("atherosclerosis", "atherosclerotic","atherogenic"))

#Cardiovascular. Search keyword based on MeSH Unique ID for Cardiovascular: D002319

During manual curation, we focussed on the connection of diabetes with cardiovascular (for example, cardiovascular(CVD)). Disambiguation of abbreviations were carried out using the head_abbrev() function of the R package pubmed.mineR. As an example - *Mutation in intron 2 of BCO2 gene is also supposed to be related to the expression of IL-18, a pro-inflammatory cytokine associated with obesity, cardiovascular diseases, and type 2 diabetes. PMID:27390265*

official_fn(hgnc_gene_table_1,cardio,"Cardiovascular_",c("cardiovascular","ischemic stroke","CHD","coronary heart disease",”CVD”))

alias_fn(hgnc_gene_table_1,hgnc_gene_table,cardio,"Cardiovascular_",c("cardiovascular", "ischemic stroke","CHD","coronary heart disease",”CVD”))

prevsymbol_fn(hgnc_gene_table_1,hgnc_gene_table,cardio,"Cardiovascular_",c("cardiovascular","ischemic stroke","CHD","coronary heart disease",”CVD”))

names_fn(hgnc_gene_table_1,hgnc_gene_table,cardio,"Cardiovascular_",c("cardiovascular", "ischemic stroke","CHD","coronary heart disease",”CVD”))

                                                    ********************************

In addition the functions Find_conclusion() was used to extract conclusions mentioned or in the extreme case, the entire abstract was carefully examined. In cases where the full paper was available in the Pubmed Central, the function Give_Sentences_PMC() was used to extract the relevant sentences and carefully examined.

***************************************************************************** #Source code for miRNA associated with T2DM#

miRNA .Search keyword used for miRNA were: ”miRNA” and “miR”. During manual curation we focussed on the connection of type 2 diabetes with miRNA accompanying gene targets.

As an example - *The results suggest that platelet-derived “miR-103b” could negatively regulate the expression of SFRP4 mRNA/protein in pre-DM2, indicating that “miR-103b could be a novel biomarker for the early diagnosis of DM2.PMID:25820527*

#Secondary corpus for miRNA

mirna_corpus = searchabsL(diabetes,include = c("miRNA","miR"))

#Observations containing gene symbol (official, alias, previous symbol gene name) and PMID. All results were manually examined and False positives were removed:

official_fn(hgnc_gene_table_1,mirna_corpus,"mirna_",c("miRNA","miR"))

prevsymbol_fn(hgnc_gene_table_1,hgnc_gene_table,mirna_corpus,"mirna_",c("miRNA","miR"))

names_fn(hgnc_gene_table_1,hgnc_gene_table,mirna_corpus,"mirna_",c("miRNA","miR"))

alias_fn(hgnc_gene_table_1,hgnc_gene_table,mirna_corpus,"mirna_",c("miRNA","miR"))

******************************************************************************

#Source code for gene data mining for T2D associated with six different population #

#Secondary corpus for six different population

population_china = searchabsL(diabetes,include = c("Chinese","China"))

population_mexican = searchabsL(diabetes,include = c("Mexican","Mexico"))

population_indian = searchabsL(diabetes,include = c("Indian","India"))

population_european = searchabsL(diabetes,include = c("European","Europe"))

population_japan = searchabsL(diabetes,include = c("Japan","Japanese"))

population_American = searchabsL(diabetes,include = c("American","America"))

#Observations containing gene symbol (official, alias, previous symbol gene name) and PMID were extracted. All results were manually examined and False positives were removed:

#Chinese. Search keywords used for Chinese were:”Chinese” and “China”.During manual curation we focussed on the T2D genes studied in the chinese population along with the SNPs IDs, risk allele, chromosome no., chromosome position, functional class, p-value and Odds-ratio.

As an example - *The GCKR rs780092 variant showed opposite-directional associations with type 2 diabetes and hypertriacylglycerolaemia in a Chinese population.PMID:26515422*

official_fn(hgnc_gene_table_1,population_china,"chinese",c("chinese","China"))

prevsymbol_fn(hgnc_gene_table_1,hgnc_gene_table,population_china,"chinese",c("chinese", "China"))

alias_fn(hgnc_gene_table_1,hgnc_gene_table,population_china,"chinese",c("chinese","China"))

names_fn(hgnc_gene_table_1,hgnc_gene_table,population_china,"chinese",c("chinese","China"))

#Japanese. Search keyword used for Japanese were:”Japanese” and “Japan”.During manual curation we focussed on the T2D genes studied in the Japanese population along with the SNPs IDs, risk allele, chromosome no., chromosome position, functional class, p-value and Odds-ratio.

As an example - *Our results indicate that effects of the six SNP loci identified in  the transethnic GWAS meta-analysis are not major among the Japanese, although SNPs in POU5F1 and MPHOSPH9 loci may have some effect on susceptibility to type 2 diabetes in this population. PMID:27115357*

official_fn(hgnc_gene_table_1,population_japan,"japanese",c("Japanese","Japan"))

prevsymbol_fn(hgnc_gene_table_1,hgnc_gene_table,population_japan,"japanese",c("Japanese", "Japan"))

alias_fn(hgnc_gene_table_1,hgnc_gene_table,population_japan,"japanese",c("Japanese","Japan"))

names_fn(hgnc_gene_table_1,hgnc_gene_table,population_japan,"japanese",c("Japanese","Japan"))

#American. Search keyword used for American were:”American” and “America”. During manual curation we focussed on the T2D genes studied in the American population along with the SNPs IDs, risk allele, chromosome no., chromosome position, functional class, p-value and Odds-ratio.

As an example - *Disruption of TBC1D4 is common among North American Inuit, resulting in exclusively elevated postprandial glucose.PMID:27561922*

official_fn(hgnc_gene_table_1,population_American,"american",c("American","America"))

prevsymbol_fn(hgnc_gene_table_1,hgnc_gene_table,population_American,"american",c("American", "America"))

alias_fn(hgnc_gene_table_1,hgnc_gene_table,population_American,"american",c("American", "America"))

names_fn(hgnc_gene_table_1,hgnc_gene_table,population_American,"american",c("American", "America"))

#Indian. Search keyword used for Indian were:”Indian” and “India”.During manual curation we focussed on the T2D genes studied in the Indian population along with the SNPs IDs, risk allele, chromosome no., chromosome position, functional class, p-value and Odds-ratio.

As an example - *These data suggest that the allele and genotypes of SLC22A1 rs622342 gene polymorphism were  associated with the therapeutic efficacy of metformin in South Indian patients with T2DM. PMID:25492374*

official_fn(hgnc_gene_table_1,population_indian,"indian",c("Indian","India"))

prevsymbol_fn(hgnc_gene_table_1,hgnc_gene_table,population_indian,"indian",c("Indian","India"))

alias_fn(hgnc_gene_table_1,hgnc_gene_table,population_indian,"indian",c("Indian","India"))

names_fn(hgnc_gene_table_1,hgnc_gene_table,population_indian,"indian",c("Indian","India"))

#Mexican. Search keyword used for Mexican were:”Mexican” and “Mexico”.During manual curation we focussed on the T2D genes studied in the Mexican population along with the SNPs IDs, risk allele, chromosome no., chromosome position, functional class, p-value and Odds-ratio.

As an example - *The R230C ABCA1 variant is associated with type 2 diabetes, particularly of early onset, in the Mexican-Mestizo population. PMID:18003760*

official_fn(hgnc_gene_table_1,population_mexican,"mexican",c("Mexican","Mexico"))

prevsymbol_fn(hgnc_gene_table_1,hgnc_gene_table,population_mexican,"mexican",c("Mexican", "Mexico"))

alias_fn(hgnc_gene_table_1,hgnc_gene_table,population_mexican,"mexican",c("Mexican", "Mexico"))

names_fn(hgnc_gene_table_1,hgnc_gene_table,population_mexican,"mexican",c("Mexican", "Mexico"))

#European. Search keyword used for European were:”European” and “Europe”.During manual curation we focussed on the T2D genes studied in the European population along with the SNPs IDs, risk allele, chromosome no., chromosome position, functional class, p-value and Odds-ratio.

As an example - *The G-allele of TP53 rs1042522 is associated with an increased prevalence of type 2 diabetes in a combined analysis of 55,521 Europeans. PMID:21283750*

official_fn(hgnc_gene_table_1,population_european,"european",c("European","Europe"))

prevsymbol_fn(hgnc_gene_table_1,hgnc_gene_table,population_european,"european",c("European", "Europe"))

alias_fn(hgnc_gene_table_1,hgnc_gene_table,population_european,"european",c("European", "Europe"))

names_fn(hgnc_gene_table_1,hgnc_gene_table,population_european,"european",c("European", "Europe"))

****************************************************

******************************************************************************

#Source Code to check false positive data.

96 genes which were described to be strongly associated with any of the 5 given complications in T2DM.We examined whether published reports emerged refuting their associative assertions. Same protocol was followed as described in the source code below and then using pattern matching we selected the sentences with the word *not* in them.

positive_hgnc_gene_table = read.xlsx("positive_hgnc_table.xlsx") # Reading in HGNC gene table containing 96 positive genes

positive_hgnc_gene_table_1 = hgnc_gene_table[,-1] #Removing the HGNC ids column

#Observations containing gene symbol (official, alias, previous symbol gene name) and PMID were extracted.

official_fn(positive_hgnc_gene_table_1,diabetes,"diabetes_",c("atherosclerosis”,”cardiovascular”,”neuropathy”,”nephropathy”,”retinopathy”))

alias_fn(positive_hgnc_gene_table_1,positive_hgnc_gene_table,diabetes,"diabetes_",c("atherosclerosis”,”cardiovascular”,”neuropathy”,”nephropathy”,”retinopathy”))

prevsymbol_fn(positive_hgnc_gene_table_1,positive_hgnc_gene_table,diabetes,"diabetes_", c("atherosclerosis”,”cardiovascular”,”neuropathy”,”nephropathy”,”retinopathy”))

names_fn(positive_hgnc_gene_table_1,positive_hgnc_gene_table,diabetes,"diabetes_", c("atherosclerosis”,”cardiovascular”,”neuropathy”,”nephropathy”,”retinopathy”))

#Negative sentences containing the word “NOT” were extracted using grep function in R.

positive_fullname = readLines("positive_official/positive_prevsymbol/positive_alias/positive_names/") #read output files from four functions used above #

positive_fullname_names_grep = grep(" not ", positive_fullname_names, fixed=TRUE) #grep sentences containing “not” term. Note one space prefixed and suffixed to the word not in order to ensure specificity.

new_names_sentence = positive_fullname_names[positive_fullname_names_grep]

new_names_temp = 1-(positive_fullname_names_grep)

write.table(new_names_temp, "new_names_temp.txt")

new_names_temp1 = scan("new_names_temp.txt", what = "numeric")

new_names_pmids = positive_fullname_names[as.numeric(new_names_temp1)]

merged_new_names = cbind(new_names_sentence, new_names_pmids)

write.table(merged_new_names, "merged_new_names.txt")  # final output file containing PMID and observations having “NOT” term#

To check whether the genes were studied further, the same were searched in our database which gave affirmative results that was previously negative as described below.

An example of negative sentence - The Ala54Thr polymorphism of the FABP2 gene is not associated with CHD, markers of the metabolic syndrome, or the fatty acid profile of serum lipids in Finnish CHD patients. PMID-12189904

An example of positive sentence - FABP2 confers susceptibility to renal disease in type 2 diabetic patients. PMID-16249461

***********************************************************

#Example Script for gene expression analysis of T2D genes of T2DiACoD database#

*****************************************LOAD LIBRARIES******************************************

library(affy)   #####to read raw affymetrix(.CEL file####

library(limma)  ###### differential expression #######

library(gplots) ##### heatmap generation #######

library(WGCNA)  ##### to average the values of gene duplicates #######

library(AnnotationDbi)

library(GEOquery)

*******************************************READ DATA*********************************************

data=ReadAffy()

eset=rma(data,normalize=FALSE)

exp=eset@assayData$exprs

colnames(exp)=c("ND_1","ND_2","ND_3","ND_4","D_1","D_2","D_3","D_4","D_5","D_6","D_7","D_8","D_9")

tiff(filename="GSExxxx_GPLxx.tiff", bg="white",height=10,width=14,units="in",res=300)

boxplot(exp)

dev.off()

nrow(exp)

*******************************************NORMALIZATION******************************************

expz=NULL;expmat2 = NULL; for (i in 1:13){MAD=mad(exp[,i],na.rm=TRUE);medx= median(exp[,i], na.rm=TRUE);expz= lapply(exp[,i], function(x){z=(0.6745*(x-medx))/MAD; return(z)}); expz1 = unlist(expz);expmat = matrix(expz1,nrow= 22283, ncol = 1, byrow=T); expmat2 = cbind(expmat2,expmat)}

colnames(expmat2)=c("ND_1","ND_2","ND_3","ND_4","D_1","D_2","D_3","D_4","D_5","D_6","D_7","D_8","D_9")

rownames(expmat2)=rownames(exp)

tiff(filename="GSExxxxx_GPLxx.tiff", bg="white",height=10,width=14,units="in",res=300)

boxplot(expmat2)

dev.off()

****************************TO GET GPL FILE FOR GENE SYMBOL*******************************

gplxx = getGEO('GPLxx', destdir=".")

gplxx = getGEO(filename = 'GPLxx.soft')

gplxx_01 = Table(gplxx)

aa=unlist(lapply(rownames(expmat2),function(x){z=which(x==gplxx$ID);return(z)}));

gene_symbol=gplxx_01$Gene.Symbol[aa];

************************* TO AVERAGE THE DUPLICATE ROWS *********************************

tempa=collapseRows(expmat2, rowGroup=gene_symbol,rowID=rownames(expmat2), method="Average")

avg_expmat2=tempa$datETcollapsed

******************** TO FIND THE DIFFERENTIAL EXPRESSED GENES ************************

design=model.matrix(~ -1 + factor(c(1,1,1,1,2,2,2,2,2,2,2,2,2)))

colnames(design)=c("group1","group2")

contrast=makeContrasts(group2-group1,levels=design)

fit=lmFit(avg_expmat2,design)

fit1=contrasts.fit(fit,contrast)

fit2=eBayes(fit1)

tab=topTable(fit2, number = 22283, adjust.method="fdr")

test=rownames(tab)

**************************** TO MATCH OUR LIST OF GENES ************************************

all_genes_t2diacod = readLines("T2D genes list")

all_genes_t2diacod = unique(all_genes_t2diacod)

match_genes=NULL;

for(i in 1:length(all_genes_t2diacod)){

a=which(all_genes_t2diacod[i]==as.character(test))

match_genes=rbind(match_genes, tab[a,])}

a=match_genes[which(match_genes$P.Value <= 0.05),]

****************** TO FIND VALUES OF DIFFERENTIAL EXPRESSED GENES *************

test1 = rownames(a)

dd=NULL

for(i in 1:length(test1)){

dd=append(dd, which(test1[i]==rownames(avg_expmat2)))}

dif_exp=avg_expmat2[dd,]

***************************************** SAVE OUTPUT ******************************************

write.csv(match_genes, "match_genes.csv")

write.csv(a, "match_genes_pval0.05.csv")

write.csv(dif_exp, "differential_expression_match_genes.csv")

******************************************** HISTOGRAM********************************************

for(i in 1:length(dif_exp)){

jpeg(file=paste(rownames(dif_exp)[i],".jpeg",sep=""),width=800, height=500, units='px')

barplot(dif_exp[i,], col=c("blue","blue","blue","blue","red","red","red","red","red","red","red","red","red"),border=TRUE,axes=TRUE, axis.lty=1,las=2,cex.names=0.7,xlab="Samples", ylab="Gene expression (MAD-Z score)" ,main=rownames(dif_exp)[i])

dev.off() }

args.legend = list(title = "Condition", x = "topright", cex = .7)

********************************************************************************************************
